# ATM-mediated DNA double-strand break response facilitated oncolytic Newcastle disease virus replication and promoted syncytium formation in tumor cells

**DOI:** 10.1371/journal.ppat.1008514

**Published:** 2020-06-01

**Authors:** Shanhui Ren, Zaib Ur Rehman, Bo Gao, Zengqi Yang, Jiyong Zhou, Chunchun Meng, Cuiping Song, Venugopal Nair, Yingjie Sun, Chan Ding

**Affiliations:** 1 Department of Avian Infectious Diseases, Shanghai Veterinary Research Institute. Chinese Academy of Agricultural Science, Shanghai, P.R. China; 2 College of Veterinary Medicine, Northwest A&F University, Yangling, Shaanxi, P.R. China; 3 Key Laboratory of Animal Virology of Ministry of Agriculture, Department of Veterinary Medicine, Zhejiang University, Hangzhou, Zhejiang, China; 4 Avian Oncogenic viruses group, UK-China Centre of Excellence on Avian Disease Research, The Pirbright Institute, Pirbright, Guildford, Surrey, United Kingdom; 5 Jiangsu Co-innovation Center for Prevention and Control of Important Animal Infectious Disease and Zoonoses, Yangzhou University, Yangzhou, Jiangsu, P.R. China; Memorial Sloan-Kettering Cancer Center, UNITED STATES

## Abstract

Deoxyribonucleic acid (DNA) damage response (DDR) is the fundamental cellular response for maintaining genomic integrity and suppressing tumorigenesis. The activation of ataxia telangiectasia-mutated (ATM) kinase is central to DNA double-strand break (DSB) for maintaining host-genome integrity in mammalian cells. Oncolytic Newcastle disease virus (NDV) can selectively replicate in tumor cells; however, its influence on the genome integrity of tumor cells is not well-elucidated. Here, we found that membrane fusion and NDV infection triggered DSBs in tumor cells. The late replication and membrane fusion of NDV mechanistically activated the ATM-mediated DSB pathway via the ATM-Chk2 axis, as evidenced by the hallmarks of DSBs, i.e., auto-phosphorylated ATM and phosphorylated H2AX and Chk2. Immunofluorescence data showed that multifaceted ATM-controlled phosphorylation markedly induced the formation of pan-nuclear punctum foci in response to NDV infection and F-HN co-expression. Specific drug-inhibitory experiments on ATM kinase activity further suggested that ATM-mediated DSBs facilitated NDV replication and membrane fusion. We confirmed that the Mre11-RAD50-NBS1 (MRN) complex sensed the DSB signal activation triggered by NDV infection and membrane fusion. The pharmacological inhibition of MRN activity also significantly inhibited intracellular and extracellular NDV replication and syncytia formation. Collectively, these data identified for the first time a direct link between the membrane fusion induced by virus infection and DDR pathways, thereby providing new insights into the efficient replication of oncolytic NDV in tumor cells.

## Author summary

Various viruses have evolved a plethora of strategies to manipulate DNA damage machinery and commandeer cells to maximize their own replication. On the contrary, host cells have also evolved to maintain cellular genomic stability and suppress carcinogenesis through multifaceted cellular DNA damage response (DDR). Most studies have focused on the relationship between DNA virus replication and the host DDR pathway, but few have investigated the interface between the DDR pathway and ribonucleic acid (RNA) viruses, such as oncolytic Newcastle disease virus (NDV). In the present study, we report for the first time that F-HN co-expression and NDV infection trigger ataxia telangiectasia-mutated (ATM)-dependent DSB lesions in tumor cells to promote viral replication and syncytium formation, respectively. We found that late NDV infection and membrane fusion activated the ATM-Chk2 axis in tumor cells, which expands prior understanding of the membrane fusion process and NDV infection. Our findings reveal new links among oncolytic NDV replication, membrane fusion, and DDR and deepen insights into NDV replication.

## Introduction

Deoxyribonucleic acid (DNA) damage response (DDR) is the cellular machinery that senses DNA damage and activates a signaling cascade to repair such damage [1]. Host cells have developed highly organized, coordinated regulatory DDR networks to ameliorate extrinsic and intrinsic stresses, maintain genomic integrity, and suppress tumorigenesis [2]. Genomic instability is a primary hallmark of tumorigenesis [1, 3]. DDR regulates and coordinates cellular DNA damage via a three-tiered signaling cascade from sensors to transducers, to effectors [4]. In mammalian cells, DDR is mediated primarily by three large serine and threonine kinase transducers [5, 6], namely, ataxia telangiectasia-mutated (ATM) protein kinase [7–9], ataxia telangiectasia and Rad3-related (ATR) [10, 11], and DNA-dependent protein kinase (DNA-PK) [6, 12]. These belong to the phosphatidylinositol 3-kinase-like kinase family and contain a typical domain of a phosphatidylinositol 3-kinase signature at their carboxyl termini. ATM and DNA-PKs are primarily activated by double-strand breaks (DSBs) [2], whereas ATR primarily transduces single-strand DNA breaks, including stalled replication forks [10].

In contrast to the classic signal transduction activated by the ligands of receptor kinases, the DDR signaling pathway is activated through the phosphorylation of the three-tiered DDR cascade driven by direct or indirect stress [1, 3, 13]. In all cases, each DNA damage lesion has a specific damage sensor that recognizes the damage and activates the most upstream DDR kinases to recruit mediators for the repair and induction of proliferation checkpoints. In particular, the Mre11-RAD50-NBS1 (MRN) complex senses the broken ends of DSBs and recruits the ATM kinase [7, 9, 14]. Meanwhile, ATR is recruited to single-strand DNA broken by the replication factor A through its cofactor ATR-interacting protein and is activated by binding proteins [11]. The tethering activity of the MRN complex is dispensable for ATM monomerization and activation in response to DSBs [14]. DNA-PKs are the central regulator of the non-homologous end joining-mediated repair of DSBs, which are recruited to DSBs and stabilized by the Ku70-Ku80 heterodimer [12, 15]. In response to DNA damage, hundreds of proteins are phosphorylated at the Ser/Thr-Glu motifs and additional sites in an ATM- or ATR-dependent manner [16]. After recruitment to the damage site, ATM and ATR phosphorylate a number of substrates [16]. For example, the histone H2A variant H2A.X is phosphorylated at Ser139 residue (known as γH2A.X); a process that occurs within minutes in response to DSBs [14, 17]. γH2A.X usually serves as a key DNA damage marker and mediates the subsequent accumulation of signaling proteins at DSB sites [3, 13].

Viruses are obligate intracellular parasites that must rely on host cells for their own genome replication needs [18]. Viruses have evolved a plethora of strategies to activate or inactivate cellular DDR to maximize their own replication, whereas host cells attempt to limit viral infection and maintain genomic stability [1, 6, 19–21]. Most current studies have focused on the interface between DNA virus infection [22], especially oncogenic DNA viruses, and the cellular DDR pathway. Only a few studies, however, have investigated the DDR pathway and RNA viruses [21], especially oncolytic Newcastle disease virus (NDV). NDV belongs to the genus *Avulavirus*, the family *Paramyxoviridae*, and the order *Mononegavirales* [23]. NDV is generally categorized into three pathotypes, namely, highly virulent (velogenic), intermediately virulent (mesogenic), or non-virulent (lentogenic) [24, 25]. The NDV genome is non-segmented, single-stranded, negative sense. It has a length of approximately 15 kb and encodes six proteins: nucleocapsid protein (NP), phosphoprotein (P), matrix protein (M), fusion protein (F), hemagglutinin-neuraminidase protein (HN), and polymerase protein (L) [25]. Two additional proteins, V and W, are derived from RNA editing of the P gene [26]. The NDV exerts oncolytic activity, selectively replicates in tumor cells, and induces cancer cell death through the intrinsic and/or extrinsic caspase-dependent pathway [27, 28]. NDV infects target tumor cells through HN-mediated attachment and then triggers F protein conformational change. Finally, it releases fusion peptide to fuse the viral and cellular membrane [29, 30], which generates typical syncytium lesions [31]. Considering the possible link between syncytium lesions and DNA damage, we aimed to elucidate the connections among membrane fusion, oncolytic NDV replication, and DDR in the present study. We report for the first time that oncolytic NDV infection and F-HN co-expression triggers the activation of ATM-mediated DSB signals to promote viral replication and syncytium formation in tumor cells.

## Results

### Oncolytic NDV infection and membrane fusion induced syncytium formation and disturbed the spatial arrangements of subcellular structures

**Fig 1:**
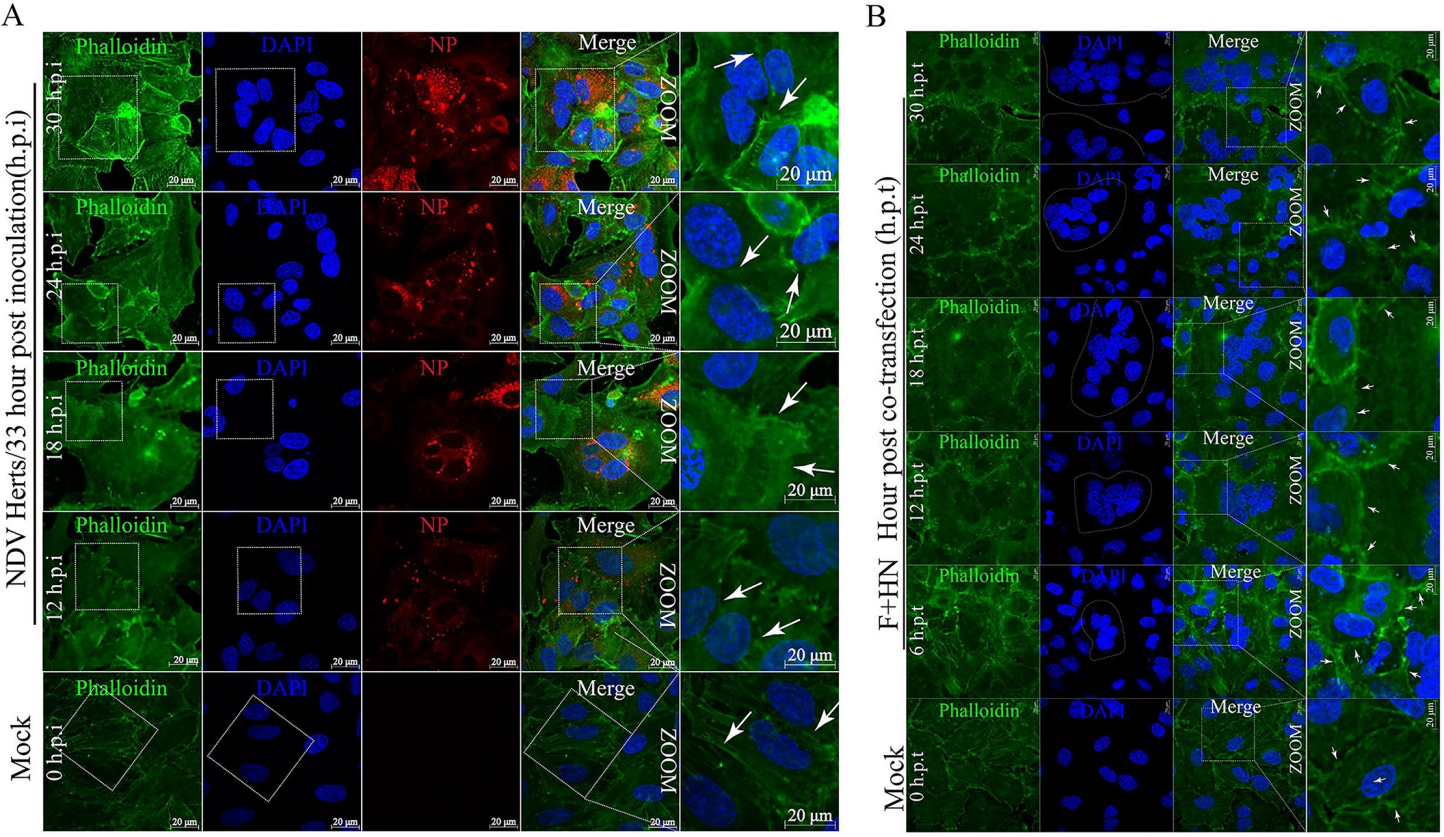
NDV infection and membrane fusion induced syncytium formation and disturbed the spatial arrangement of cellular F-actin filaments. (A) Representative images showing that oncolytic NDV infection altered the spatial arrangement of cellular F-actin filament in A549 cells. A549 cells were mock-infected and NDV-infected (Herts /33 strain) (MOI = 1), at the indicated timepoint. At 12, 18, 24, and 30 h.p.i., the coverslips were examined by IFA. After being fixed and permeabilized, the cells at a final concentration of 50 μg/ml fluorescent phalloidin conjugate the solution in PBS were inoculated for 40 min at room temperature. F-actin filament was labeled with phalloidin (green), nuclei with DAPI (blue), and NDV with the NP primary antibody (red). Arrows indicate the location of membrane fusion with nearby cells. Syncytia were marked with white dotted circles. Scale bars = 20 μm. (B) Representative images showing that F-HN co-expression altered the spatial arrangement of cellular F-actin filament in A549 cells. A549 cells were mock-transfected or co-transfected by both Flag-F and HA-HN plasmids at the indicated timepoints. At 6, 12, 18, 24, and 30 h.p.t., the coverslips were examined via IFA. Arrows indicate the location of membrane fusion with nearby cells. Syncytia were marked with dotted circles. Scale bars = 20 μm.

The typical lesion of virulent NDV infection is syncytium formation and membrane fusion in chicken embryo fibroblast (DF-1) and adenocarcinomic human alveolar basal epithelial (A549) cells [31], indicating that both cell lines can be cell models for further research. To observe the effect of syncytium formation on subcellular structures, we used phalloidin fluorescent conjugates to label endogenous F-actin filaments in A549 cells, which bind only to the polymeric and oligomeric forms of F-actin. Syncytium formation was accompanied with the spatial reorganization of subcellular structures in a time-dependent manner (Fig 1). This result suggested that the virulent NDV Herts/33 strain (hereafter denoted as NDV) infection (Fig 1A) and the dynamic co-expression of both glycoproteins (Fig 1B) disrupted cellular nucleus locations and skeletons.

Considering the typical syncytium lesions and the abnormal growth and morphology of cells, we hypothesized that NDV infection and membrane fusion may induce the formation of cellular DNA lesions accompanied with syncytium formation, thereby further triggering cellular DDR.

### NDV late replication and membrane fusion induced DNA DSBs by activating ATM-dependent signals

**Fig 2:**
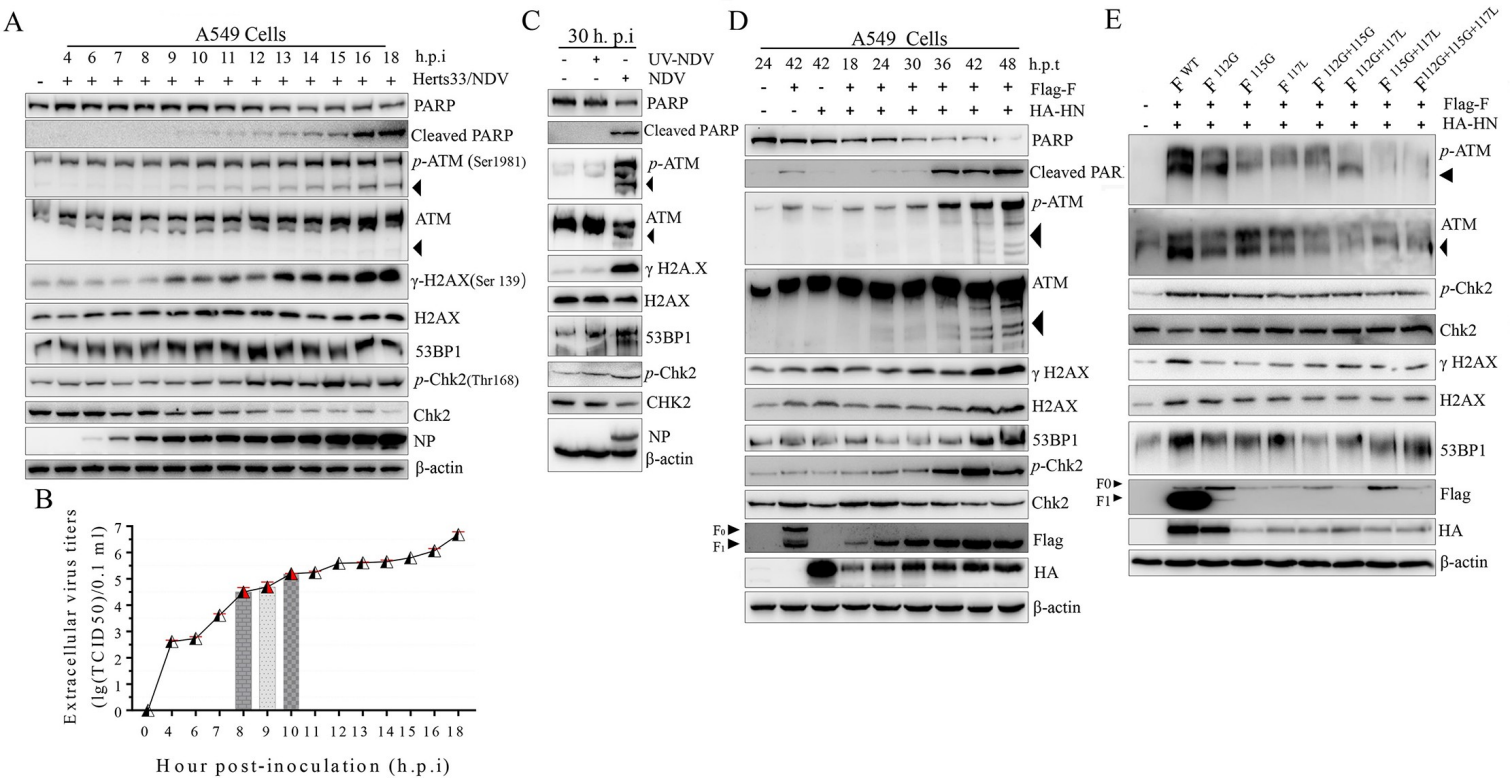
NDV late replication and membrane fusion induced DNA double strand break (DSB) through ATM kinase activation. (A) Virulent oncolytic NDV infection activated ATM-mediated DSB signaling in A549 cells. Western blot samples were prepared from A549 cells after virulent oncolytic NDV infection (Herts/33 strain) (MOI = 1) corresponding to the marked timepoints and analyzed in accordance with the procedures in the Materials and Methods section. The monomer ATM was marked with a black triangle. β-actin served as a loading control. (B) Growth curve of extracellular NDV in A549 cells. The extracellular NDV titers of culture supernatant were determined on DF-1 cells as TCID_50_ based on the Reed–Muench method. Error bars are indicated by red dashed lines. (C) NDV replication was required for ATM-mediated DSB signaling pathway activation. A549 cells were infected with virulent oncolytic NDV (Herts33/NDV strain) (MOI = 1) and UV-inactivated NDV. At 30 h.p.i., Western blot samples were collected and analyzed in accordance with the procedures in the Materials and Methods section. The monomer ATM was marked with a black triangle. (D) F and HN cooperated synergistically to activate ATM-mediated DSB in A549 cells. A549 cells were transfected with plasmids Flag-F, HA-HN, or both. Western blot samples corresponding to the marked timepoints were collected and analyzed in accordance with the procedures in the Materials and Methods section. (E) The cleavage site motif of F influenced the ATM-mediated DSB signaling pathway. In A549 cells, wild-type HN plasmids were co-transfected with wild-type F, F^112G^, F^115G^, F^117L^, F^112G+115G^, F^112G+117L^, F^115G+117L^, and F^112G+115G+117L^. At 36 h.p.t., the corresponding Western blot samples were collected and analyzed.

To confirm our hypothesis, we infected A549 cells and the human non-small cell lung cancer cells NCI-H1975 and NCI-H1299 with NDV or co-transfected F-HN, followed by DDR marker detection by Western blot analysis. The unmodified nuclear poly (ADP-ribose) polymerase (PARP) is involved in DNA repair, maintains genomic viability in response to stress agents and can be cleaved into the PARP amino-terminal DNA-binding domain (24 kDa) and the carboxy-terminal catalytic domain (89 kDa) [32]. In this study, the proteolytic cleavage of PARP was observed in A549 (Fig 2, S1 and S3 Figs, horizontal line 2), NCI-H1975 (S2A and S2C Fig, horizontal line 2), and NCI-H1299 (S2B and S2C Fig, horizontal line 2) cells in response to NDV infection and F-HN co-expression. This finding indicated caspase activation and DNA damage lesions. In response to DSBs, ATM is rapidly auto-phosphorylated on Ser1981 and dissociates from a catalytically inactive dimer into active monomers [8, 9, 13]. Here, compared with the mock-infected control, monomer ATM was activated in response to NDV infection (Fig 2A, and S1C Fig, horizontal line 4) and F-HN co-expression (Fig 2D and S2C and S3B Figs, horizontal line 4). To further confirm DSB activation during the time course of NDV infection and membrane fusion, we then examined the expression levels of ATM auto-phosphorylation on Ser 1981, an indicator of its kinase activity [8]. We found that ATM was remarkably phosphorylated in A549 cells (Fig 2, S1C and S3B Figs), NCI-H1975 (S2A and S2C Fig), and NCI-H1299 (S2B and S2C Fig) cells in response to NDV infection and F-HN co-expression compared with that in the mock control. All these outcomes were consistent with the observation from the ultraviolet (UV) exposure and etoposide treatment group (S1A Fig). ATM activation by DSB phosphorylates a number of downstream target molecules, particularly H2A.X, p53 binding protein 1 (53BP1), and Chk2 [1, 6, 13]. To further confirm the ATM-mediated DSB, we then assessed the phosphorylation level of ATM kinase downstream substrates by Western blot analysis. Compared with that of the mock control group, the expressions of the ATM-dependent phosphorylation of Chk2 (Thr 68), γ-H2A.X (Ser 139), and 53BP1 were up-regulated by late virulent NDV infection and membrane fusion in a time-dependent manner in A549 (Fig 2 and S3B Fig, horizontal lines 5 to 9), NIC-H1975 (S2A and S2C Fig), and NCI-1299 (S2B and S2C Fig) cells. Meanwhile, we observed that both velogenic (Herts/33) and lentogenic (La Sota) strains activated the ATM-mediated DSBs in A549 tumor cells (Fig 2A and 2B and S1C Fig). Moreover, compared with the La Sota group, we found that Herts/33 triggered stronger activation of ATM-dependent DSB signals, as evinced by the Western blot analysis results of total PARP, cleaved-PARP, total ATM, *p*-ATM, total H2A.X, γ-H2A.X, 53BP1, total Chk2, and *p*-Chk2 (S1C Fig, horizontal lines 1 to 9). The activation state of ATM-mediated DSBs was thus positively correlated with the virulence of NDV. Unlike the NDV group, UV-NDV could not activate the downstream substrates of ATM-mediated DSBs (Fig 2C, vertical line 2), revealing the significance of NDV lytic replication in activating ATM-dependent downstream molecules.

Furthermore, the mutation of F cleavage sites significantly impaired the signal activation of ATM-dependent downstream molecules, such as 53BP1, γ-H2A.X, and phosphorylated ATM and Chk2 (Fig 2E, vertical lines 3 to 9) compared with the wild-type F (Fig 2E, vertical line 2). Thus, the cleavage ability of F protein precursor (F_0_) was required for ATM-mediated DSB induction. Unlike the co-transfection of velogenic NDV F-HN, however, lentogenic NDV F-HN cannot activate ATM-dependent DSB signals in A549, NCI-H1975, or NCI-1299 cells (S2C Fig). This finding indicated that membrane-fusion functions on the activation of ATM-dependent DSBs, which is consistent with the results shown in Fig 2D and S3B Fig. To further elucidate whether the possible involvement of other viral structural proteins (NP, P, M, and L) or non-structural proteins (V and W) in the activation of ATM-mediated DSB signals, we next transfected Flag-NP, Flag-P, Flag-M, Flag-M, Flag-M^ΔNLS(247aa~263aa)^, Flag-V, Flag-W, Myc-L, Flag-F, HA-HN, and F-HN in A549 cells (S3A Fig). We subsequently detected signal markers of ATM-dependent DSBs through Western blot analysis (S3B Fig). Based on the Western blot analysis, we observed that only F and HN acted synergistically to activate ATM-mediated DSB signals (S3B Fig, vertical line 11) in A549 cells. Meanwhile, considering the nuclear translocation characterization of the structural M protein throughout the entire life cycle of NDV replication (S3A Fig), we comparatively determined whether the nuclear aggregation of *p*-ATM (Ser 1981), γ-H2A.X (Ser 139), 53BP1, and *p*-CHK2 (Thr 68) between wild-type M group and the deleted nuclear localization sequence (NLS) M (M^ΔNLS^) group occurred in transfected A549 cells through immunofluorescence analysis (IFA) assay. As expected, no difference was observed between the wild-type M and M^ΔNLS^ groups (S3C Fig), consistent with the Western blot results in S3B Fig (vertical lines 4 and 5). Thus, the nuclear translocation of M had no influence on the activation of ATM kinase in tumor cells.

We further evaluated the spatial redistribution of auto-phosphorylated ATM at Ser 1981 by using IFA during NDV infection and membrane fusion. Compared with nuclei barely forming bright foci in the mock group, we found that most of the infected cells induced the formation of large auto-phosphorylated foci in the nuclei in A549 (S4 Fig), NCI-H1975 (S5A Fig), and NCI-H1299 (S5B Fig) cells. Interestingly, we also found the re-localization of large bright auto-phosphorylated ATM punctum foci from nuclei to cytoplasm in partially infected tumor cells (S4 and S5 Figs), indicating that activated ATM may be involved in multiple cellular biological processes, such as selective pexophagy [33]. Taken together, these results suggested that late NDV infection and syncytium formation triggered by virulent F-HN co-transfection significantly activated the ATM-mediated DSB signaling pathway in tumor cells.

### Aggregation of γ-H2A.X and p53 binding protein 1 (53BP1) foci was induced by oncolytic NDV infection and membrane fusion

**Fig 3:**
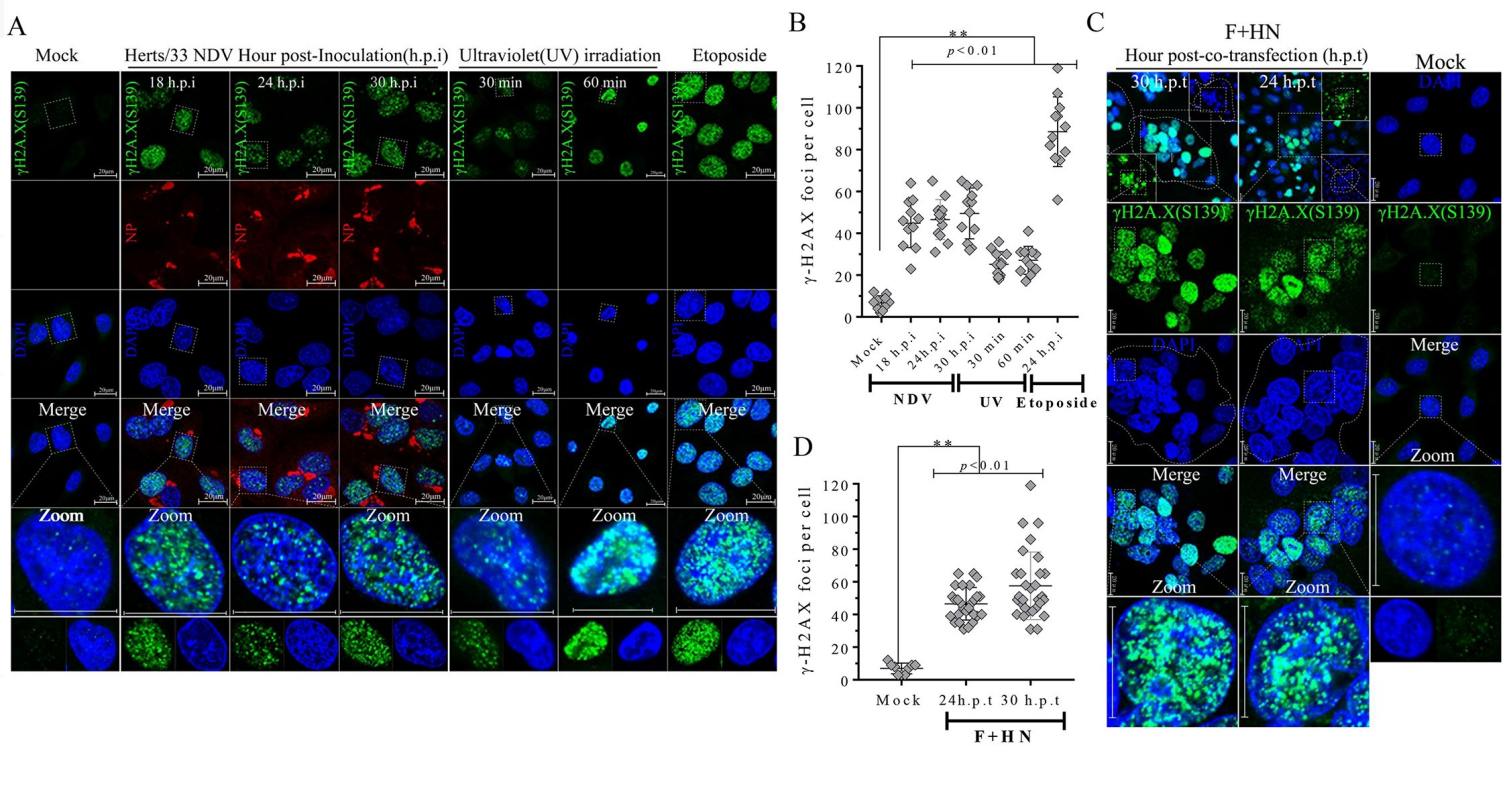
Aggregation of γ-H2A.X punctuate foci in nuclei were induced by virulent NDV infection and membrane fusion. (A) Representative images showing that virulent oncolytic NDV infection triggered the nuclear aggregation of γ-H2A.X in A549 cells. A549 cells were mock-infected, NDV-infected (MOI = 1), inoculated for 18, 24, and 30 h, UV exposed for 30 and 60 min, and treated with etoposide at a final concentration of 80 μm for 24 h. After treatment, the A549 cells were collected, fixed, and visualized by IFA. The UV and etoposide treatment groups were used as positive controls. γ-H2A.X (green); Nuclei (blue); NDV (red). Scale bars = 20 μm. (B) Statistical analysis of the nuclear aggregation punctate foci number of γH2AX in response to virulent oncolytic NDV infection in A549 cells. Numerical data were the average number of punctate foci in different microscope fields in at least three independent experiments. Significance was analyzed using two-tailed Student’s t-test. NS, *p* > 0.05; *, *p* < 0.05; **, *p* < 0.01; ***, *p* < 0.001. The UV and etoposide groups served as positive controls. (C) Representative images showing that F-HN co-expression triggered the nuclear aggregation of γ-H2A.X in A549 cells. A549 cells were mock-transfected or co-transfected with both Flag-F and HA-HN plasmids at the indicated timepoints. At 24 and 30 h.p.t., the coverslips were examined by IFA. γ-H2A.X (green); Nuclei (blue); NDV (red). Scale bars = 20 μm. (D) Statistical analysis of the nuclear aggregation punctate foci number of γ-H2A.X in response to F-HN co-expression.

**Fig 4:**
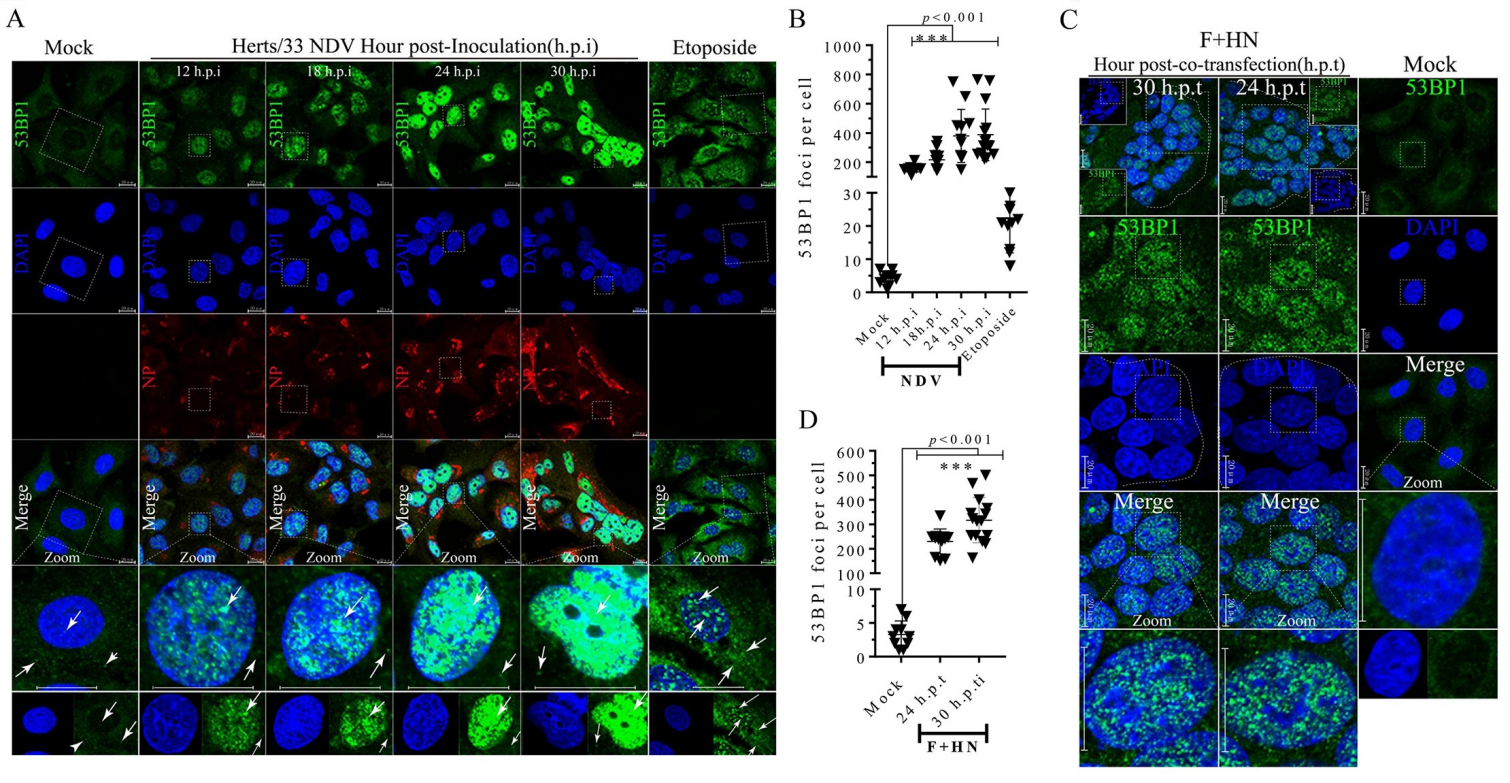
Aggregation of p53 binding protein 1 (53BP1) punctate foci in nuclei was induced by NDV infection and membrane fusion. (A) Representative images showing that virulent oncolytic NDV infection triggered nuclear aggregation of endogenous 53BP1 in A549 cells. A549 cells were mock-infected, NDV-infected (MOI = 1), inoculated for 12, 18, 24, and 30 h, and treated with etoposide at a final concentration of 80 μm for 24 h. The etoposide treatment served as the positive control. 53BP1 (green); nuclei (blue); NDV (red). Arrows indicate 53BP1 localization in the nuclei. Scale bars = 20 μm. (B) Statistical analysis of the nuclear aggregation punctate foci number of endogenous 53BP1 in response to virulent oncolytic NDV infection in A549 cells. Numerical data were the average number of punctate foci in different microscope fields in at least three independent experiments. Significance was analyzed using two-tailed Student’s t-test. NS, *p* > 0.05; *, *p* < 0.05; **, *p* < 0.01; ***, *p* < 0.001. (C) Representative images showing that F-HN co-expression triggered nuclear aggregation of endogenous 53BP1 in A549 cells. A549 cells were mock transfected or co-transfected with both Flag-F and HA-HN plasmids at the indicated time. At 24 and 30 h.p.t., the coverslips were examined by IFA. 53BP1 (green); nuclei (blue); NDV (red). Arrows indicate 53BP1 localization in the nuclei. Scale bars = 20 μm. (D) Statistical analysis of the nuclear aggregation punctate foci number of 53BP1 in response to F-HN co-expression.

**Fig 5:**
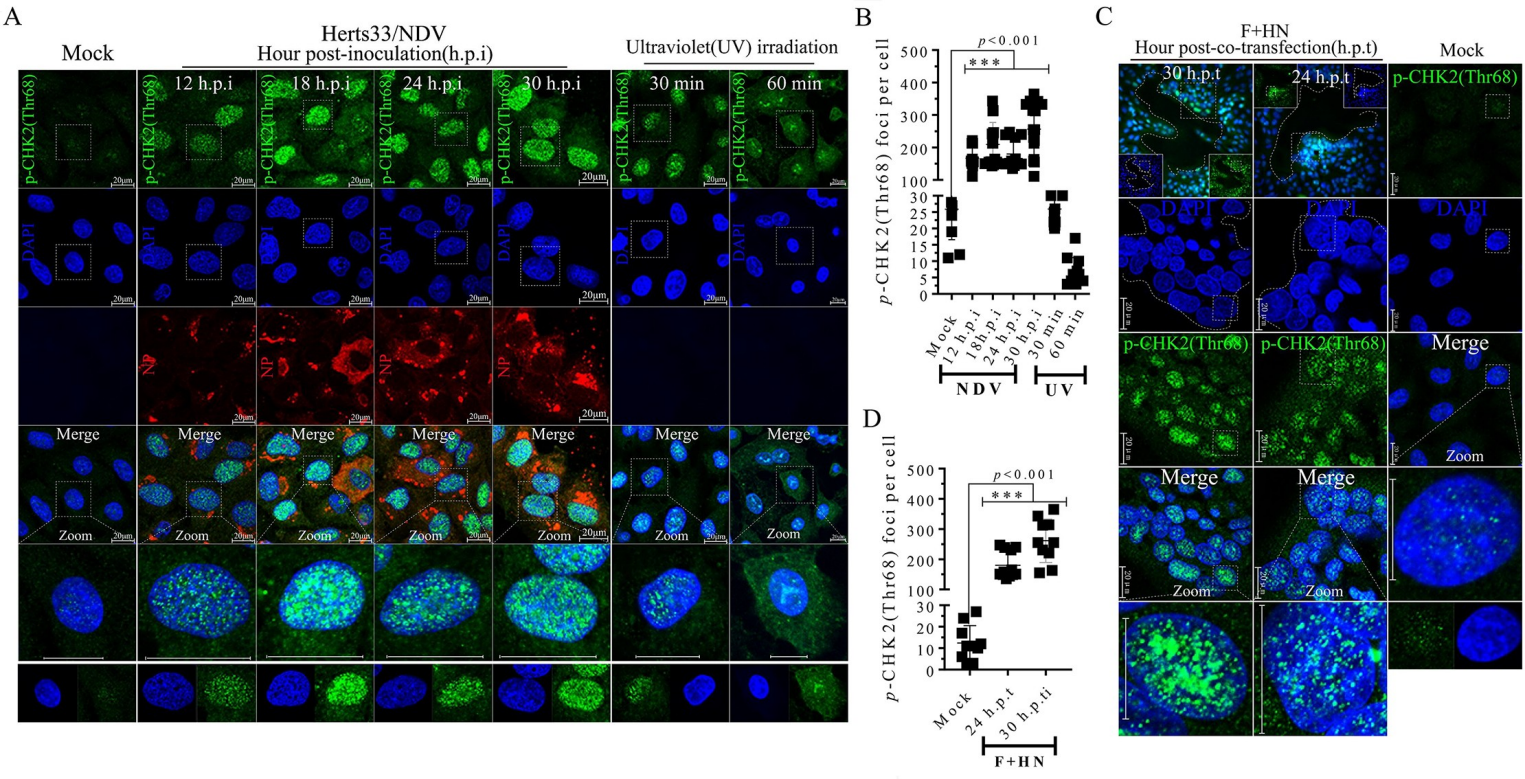
NDV late infection and membrane fusion activated the ATM-Chk2 axis in A549 cells. (A) Representative images showing that virulent oncolytic NDV infection triggered nuclear aggregation of phosphorylated Chk2 on Thr48 in A549 cells. A549 cells were mock-infected and NDV-infected (MOI = 1) for 12, 18, 24, and 30 h, and UV exposed for 30 and 60 min. After treatment, the A549 cells were collected, fixed, and visualized by IFA. The UV-exposure group served as the positive control. Phosphorylated Chk2 (green); nuclei (blue); NDV (red). Scale bars = 20 μm. (B) Statistical analysis of the nuclear aggregation punctate foci numbers of phosphorylated Chk2 on Thr 48 in response to virulent oncolytic NDV infection in A549 cells. Numerical data were the average number of punctate foci in different microscope fields in at least three independent experiments. Significance was analyzed using two-tailed Student’s t-test. The UV-exposure group served as the positive control. NS, *p* > 0.05; *, *p* < 0.05; **, *p* < 0.01; ***, *p* < 0.001. (C) Representative images showing that F-HN co-expression triggered nuclear aggregation of phosphorylated Chk2 on Thr 48 in A549 cells. A549 cells were mock transfected or co-transfected with both Flag-F and HA-HN plasmids at the indicated time. At 24 and 30 h.p.t., coverslips were examined by IFA. Phosphorylated Chk2 (green); nuclei (blue); NDV (red). Scale bars = 20 μm. (D) Statistical analysis of the nuclear aggregation punctate foci number of phosphorylated Chk2 on Thr 48 in response to F-HN co-expression.

Following the initial activation of ATM by DSBs, ATM triggers a cascade of downstream DDR events on chromatin flanking DSBs [3]. We subsequently assessed the influence of NDV infection and co-expression of F-HN on the localization of γ-H2A.X to further confirm the aforementioned DSB phenomenon. Compared with that of the mock control in A549 and NCI-H1975 cells, more and larger bright punctuate foci of γ-H2A.X significantly aggregated throughout the nuclei in response to NDV infection (Fig 3A, vertical lines 2 to 4 and Fig 3B) and syncytium formation (Fig 3C, vertical lines 1 to 2, and Fig 3D) in A549 and NCI-1975 (S6A Fig) cells. This finding suggested the robust induction of the γ-H2A.X signaling cascade in an ATM-dependent manner. Cumulative evidence suggests that γ-H2A.X around DSBs triggers cascades to promote the recruitment of 53BP1, a member of the BRCT protein family [17]. We subsequently examined the spatial distribution of endogenous 53BP1 in response to NDV infection and membrane fusion in A549 and NCI-H1975 cells. Compared with the mock control, many large 53BP1 nuclei aggregated in a time-dependent manner in response to virulent NDV infection (Fig 4A, vertical lines 2 to 5, and Fig 4B) and syncytium formation (Fig 4C, vertical lines 1 to 2, and Fig 4D) in A549 and NCI-1975 (S6B Fig) cells, indicating the activation state of endogenous 53BP1. In summary, ATM-mediated DSB signals subsequently activated the downstream substrates H2A.X and 53BP1 during late NDV infection and membrane fusion. Based on the results of IFA (Figs 4 and 5 and S6 Fig) and Western blot analysis (Fig 2 and S1 to S3 Figs), we speculated that γ-H2A.X is involved in the recruitment of 53BP1 to DSB sites and sequential phosphorylation events following virulent NDV infection and syncytium formation.

### Late NDV infection and membrane fusion activated the ATM-Chk2 axis in tumor cells

ATM kinase directly phosphorylates Chk2 on specific threonine 68 via the ATM-Chk2 axis, which is completely restricted to DSB sites before it can spread throughout a nucleus as an active kinase [3, 34, 35]. We subsequently investigated the impact of activated ATM on Chk2 phosphorylation in Thr 68 during NDV infection and membrane fusion in A549 and NCI-H1975 cells. Compared with the mock control, NDV infection (Fig 5A, vertical lines 2 to 5 and Fig 5B) and F-HN co-expression (Fig 5C, vertical lines 1 to 2 and Fig 5D) significantly triggered the aggregation of punctum foci of phosphorylated Chk2 in the nuclei of A549 and NCI-H1975 cells (S6C Fig). This finding was consistent with the positive UV exposure and etoposide treatment groups, suggesting the activation of downstream kinase Chk2 via the ATM-Chk2 axis in response to NDV infection and syncytium formation. Based on IFA (Fig 5 and S6C Fig) and Western blot analysis (Fig 2 and S1 to S3 Figs), we speculated that ATM kinase activated the endogenous Chk2 via the ATM-Chk2 axis and that, in turn, the activated Chk2 coordinated the candidate transmitter of the DSB signals to damaged-DNA sites in response to virulent NDV infection and syncytium formation.

### Inhibition of ATM kinase activity suppressed the NDV replication and syncytium formation

**Fig 6:**
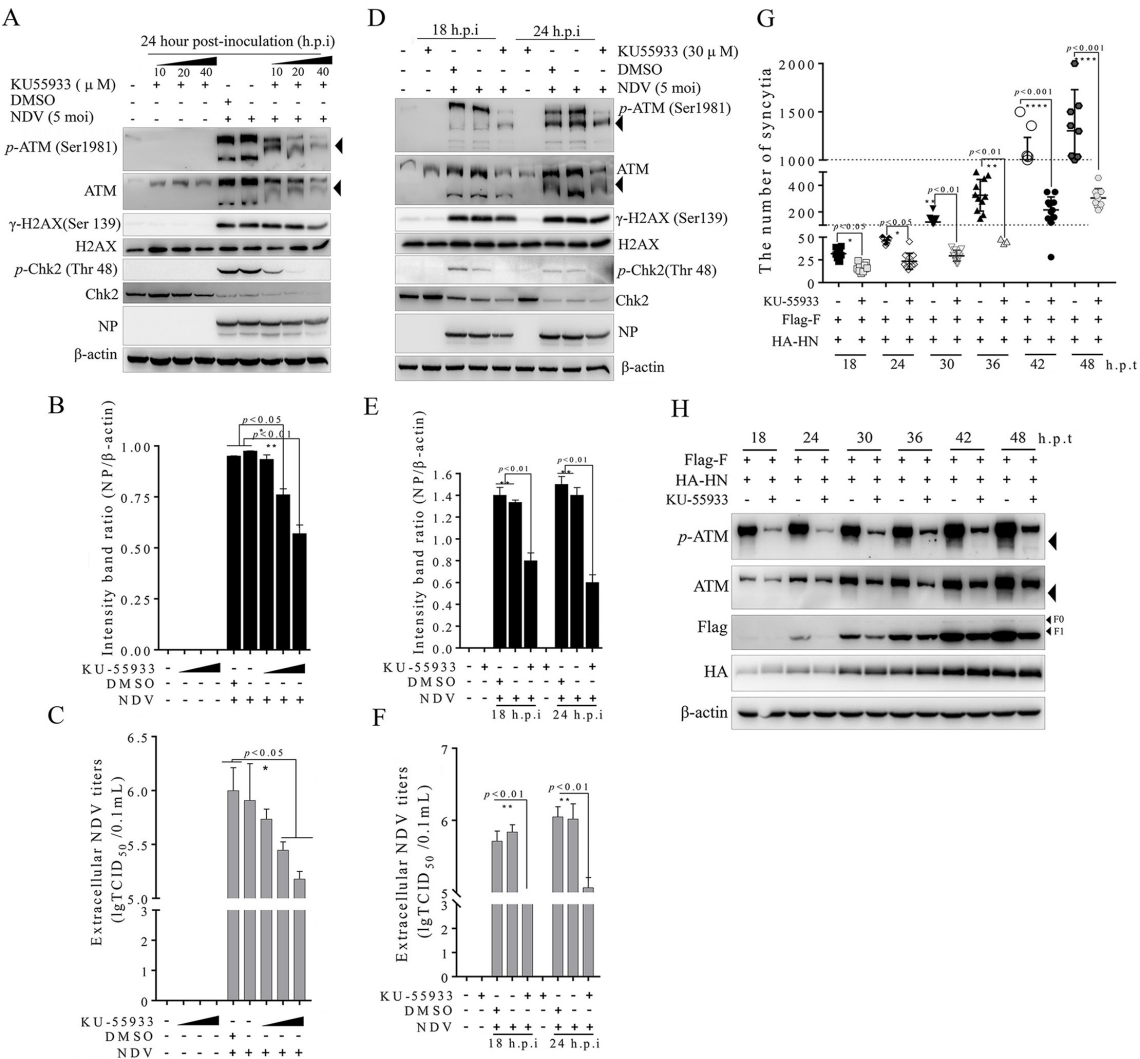
Inhibition of ATM kinase activity suppressed NDV replication and syncytium formation. (A) The ATM-dependent signaling pathway was required for NDV intracellular replication in a dose-dependent manner. A549 cells were pretreated at a working concentration of KU55933 at 10, 20, and 40 μm for 1 h prior to NDV infection. A549 cells were mock-infected, NDV-infected (MOI = 5) for 24 h. At 24 h.p.i., Western blot samples were prepared and analyzed in accordance with the procedures in the Materials and Methods section. The monomer ATM was marked with a black triangle. β-actin served as a loading control. (B) The intensity band ratio of intracellular NDV NP to β-actin. Data were presented as means from three independent statistical experiments. The intensities of protein bands were quantified using Image J. Significance was analyzed using a one-tailed Student’s t-test. NS, *p* > 0.05; *, *p* < 0.05; **, *p* < 0.01; ***, *p* <0.001. (C) The ATM-dependent signaling pathway was required for NDV extracellular replication in a dose-dependent manner. A549 cells were mock-infected, NDV-infected (MOI = 5) for 24 h. At 24 h.p.i., the cell-culture supernatants were collected and the extracellular NDV titers were determined on DF-1 cells as TCID_50_ based on the Reed-Muench method. Significance was analyzed using a two-tailed Student’s t-test. NS, *p* > 0.05; *, *p* < 0.05; **, *p* < 0.01; ***, *p* < 0.001. (D) The ATM-dependent signaling pathway was required for NDV intracellular replication in a time-dependent manner. A549 cells were pretreated at a working concentration of KU55933 at 30 μm for 1 h prior to NDV infection. A549 cells were mock-infected and NDV-infected (MOI = 5) for 18 and 24 h. At 18 and 24 h.p.i., Western blot samples were prepared and analyzed. (E) The intensity band ratio of intracellular NDV NP to β-actin. The intensities of protein bands were quantified using Image J. (F) The ATM-dependent signaling pathway was required for NDV extracellular replication in a time-dependent manner. A549 cells were mock-infected and NDV-infected (MOI = 5) for 18 h and 24 h. At 18 and 24 h.p.i., the cell-culture supernatants were collected and the extracellular NDV titers were determined. (G) Statistical analysis of the effect of ATM inhibitor KU55933 on syncytium formation in A549 cells. A549 cells were pretreated at a working concentration of KU55933 at 30 μm for 1 h prior to transfection and co-transfected with both Flag-F and HA-HN plasmids in the absence or presence of KU55933. Syncytium formation was observed on the basis of the number of nuclei. Numerical data were the average numbers of syncytia in different microscope fields in at least three independent experiments. Significance was analyzed using two-tailed Student’s t-test. NS, *p* > 0.05; *, *p* < 0.05; **, *p* < 0.01; ***, *p* < 0.001. (H) Western blot analysis of the effect of ATM inhibitor KU55933 on syncytium formation in A549 cells. A549 cells were pretreated at a working concentration of KU55933 at 30 μm for 1 h prior to transfection and co-transfected with both Flag-F and HA-HN plasmids. Western-blot samples corresponding to the marked timepoints were collected and analyzed.

To further determine the effect of the ATM-dependent DSB signaling pathway on NDV replication and membrane fusion, we blocked ATM kinase activity by using KU-55933[36], a specific pharmacological inhibitor. As shown in Fig 6A and 6D, the total level of endogenous ATM and phosphorylated ATM in the NDV-infected group was inhibited when treated with KU55933 compared with that of the dimethylsulfoxide (DMSO) control group. This result suggested that the specific inhibitor KU55933 successfully blocked the ATM kinase activity triggered by NDV infection and severely diminished the protein levels of γ-H2AX (Fig 6A and 6D, horizontal line 3), and *p*-Chk2 (Fig 6A and 6D, horizontal line 5) in a time- and dose-dependent manner. This finding suggested that ATM kinase activity was essential for the activation of ATM-dependent downstream molecules. The inhibition of ATM kinase activity suppressed intracellular viral titers as indicated by the structural NP of NDV (Fig 6A and 6D, horizontal line 7, and Fig 6B and 6E), as well as extracellular viral titers as measured using the median tissue culture infective dose (TCID_50_) method (Fig 6C and 6F) compared with the DMSO treatment. This outcome suggested that ATM-mediated DSBs were required for NDV replication. Similarly, KU-55933 significantly blocked syncytium formation in a time-dependent manner compared with the F-HN co-expression group (Fig 6G). This outcome was consistent with the Western blot results (Fig 6H), suggesting that ATM kinase was required for membrane fusion. Taken together, these findings indicated that ATM-mediated DSB signal activation played a vital role in NDV replication and syncytium formation.

### Virulent NDV infection and membrane fusion activated MRN complex sensors in tumor cells

**Fig 7:**
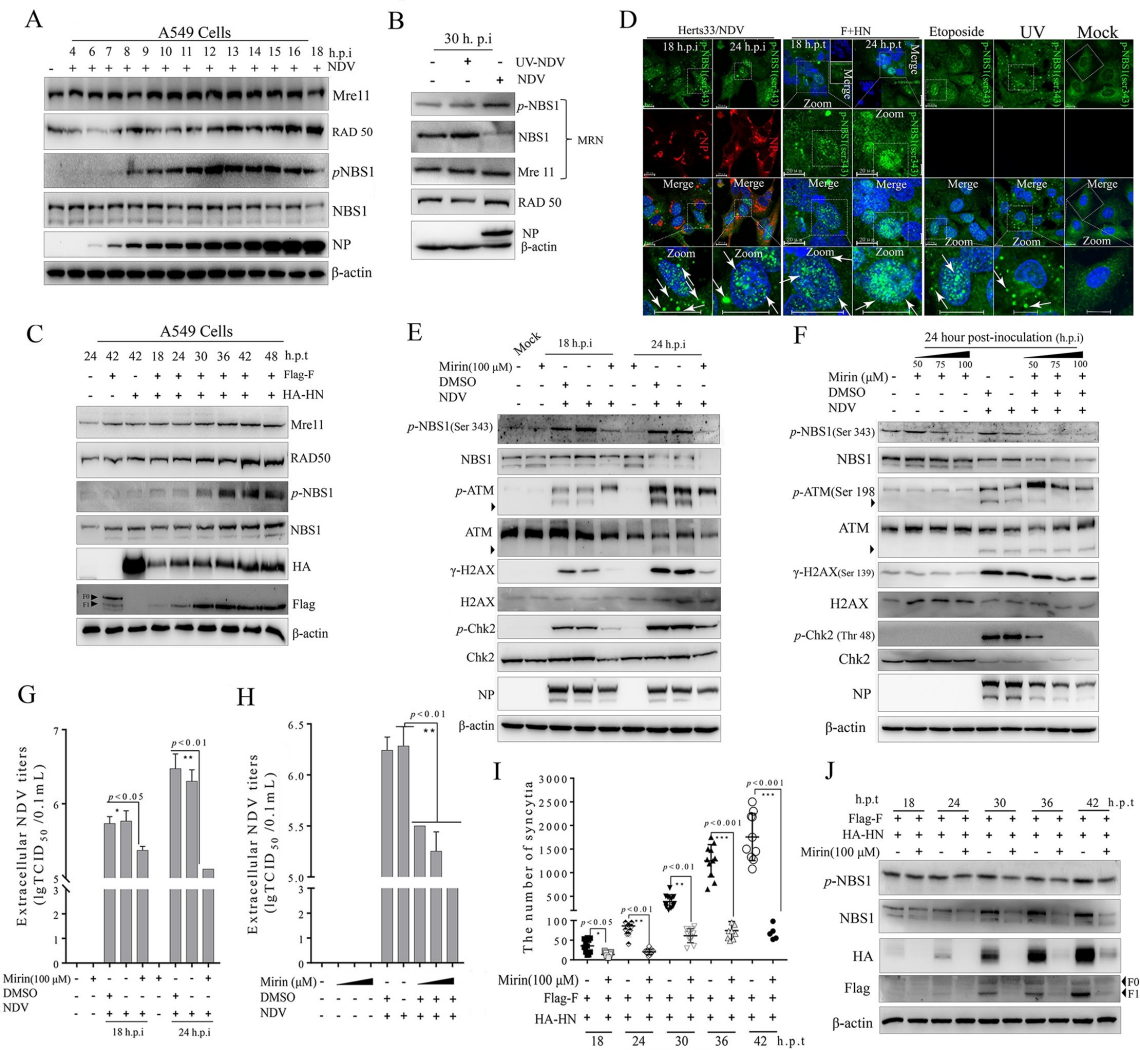
Virulent NDV infection and F-HN co-expression activated the Mre11-Rad50-NBS1 (MRN) complex sensor in A549 cells. (A) Virulent oncolytic NDV infection activated the MRN sensor of ATM-dependent DSB signaling in A549 cells. Western blot samples were prepared from A549 cells after virulent oncolytic NDV infection (Herts 33/NDV strain) (MOI = 1) corresponding to the marked timepoints, and analyzed in accordance with the procedures in the Materials and Methods section. β-actin served as a loading control. (B) NDV replication was essential for the activation of MRN sensor. A549 cells were inoculated with virulent oncolytic NDV (Herts33/ NDV) (MOI = 1) and UV-inactivated NDV. At 30 h.p.i., Western blot samples were collected and analyzed in accordance with the procedures in the Materials and Methods section. (C) F with HN cooperated synergistically to activate the MRN sensor of ATM-mediated DSB signaling in A549 cells. A549 cells were transfected with the plasmids Flag-F, HA-HN, or both. Western blot samples corresponding to the marked timepoints were collected and analyzed in accordance with the procedures in the Materials and Methods section. (D) Representative images showing that virulent oncolytic NDV infection and F-HN co-expression triggered nuclear aggregation of phosphorylated NBS1 on Ser343 in A549 cells. A549 cells were mock-infected, or NDV-infected (MOI = 1) for 18 and 24 h, UV-exposed for 30 min, and treated with etoposide at a final concentration of 80 μm for 24 h. The UV and etoposide treatment groups served as the positive controls. A549 cells were co-transfected with both Flag-F and HA-HN plasmids at the indicated timepoints. At 18 and 24 h.p.t., coverslips were examined by IFA. *p*-NBS1 (green); nuclei (blue); NDV (red). Scale bars = 20 μm. (E) MRN complex activity was required for intracellular NDV replication in a time-dependent manner. A549 cells were pretreated with a working concentration of Mirin at 100 μm for 1 h prior to NDV infection. A549 cell were mock-infected and NDV-infected (MOI = 5) for 18 and 24 h. At 18 and 24 h.p.i., Western blot samples were prepared and analyzed in accordance with the procedures in the Materials and Methods section. The monomer ATM was marked with a black triangle. β-actin served as a loading control. (F) MRN complex activity was required for intracellular NDV replication in a dose-dependent manner. A549 cells were pretreated at a working concentration of Mirin at 50, 75, and 100 μm for 1 h prior to NDV infection and mock-infected and NDV-infected (MOI = 5) for 24 h. (G) MRN complex activity was required for NDV extracellular replication in a time-dependent manner. A549 cells were mock-infected, NDV-infected (MOI = 5) for 24 h. At 24 h.p.i., the cell culture supernatants were collected and the extracellular NDV titers were determined on DF-1 cells as TCID_50_ based on the Reed-Muench method. Significance was analyzed using a two-tailed Student’s t-test. NS, *p* > 0.05; *, *p* < 0.05; **, *p* < 0.01; ***, *p* < 0.001. (H) MRN complex activity was required for NDV extracellular replication in a dose-dependent manner. A549 cells were mock-infected and NDV-infected (MOI = 5) for 18 h and 24 h. At 18 and 24 h.p.i., the cell culture supernatants were collected and the extracellular NDV titers were determined on DF-1 cells. (I) Statistical analysis of the effect of the MRN inhibitor Mirin on syncytium formation in A549 cells. A549 cells were pretreated at a working concentration of Mirin at 100 μm for 1 h prior to co-transfection, and then co-transfected with both Flag-F and HA-HN plasmids in the absence or presence of Mirin. Syncytium formation was observed on the number of nuclei. Numerical data were the average number of syncytia in different microscope fields in at least three independent experiments. Significance was analyzed using a two-tailed Student’s t-test. NS, *p* > 0.05; *, *p* < 0.05; **, *p* < 0.01; ***, *p* < 0.001. (J) Western blot analysis of the effect of Mirin on syncytium formation in A549 cells. A549 cells were pretreated at a working concentration of Mirin at 100 μm for 1 h prior to transfection, and then transfected with both Flag-F and HA-HN plasmids. Western blot samples corresponding to the marked timepoints were collected and analyzed in accordance with the procedures in the Materials and Methods section. β-actin served as a loading control.

The MRN complex acts as a sensor of the ATM-induced DSB signaling pathway [2, 7, 14, 37], which is one of the first factors recruited to break DNA [38]. To ascertain the kinetics of ATM kinase and determine whether the MRN complex is involved in ATM-mediated DSB signaling triggered by NDV replication and F-HN co-expression, we evaluated the MRN protein levels in response to virulent NDV infection and F-HN co-expression by Western blot analysis in A549, NCI-H1975, and NCI-H1299 cells. Compared with UV and etoposide treatment (S1B Fig), NDV infection and F-HN co-expression significantly increased endogenous NBS1 phosphorylation on Ser 343 in A549 (Fig 7A and 7C and S1C Fig), NCI-H1975 (S2A and S2C Fig), and NCI-H1299 (S2B and S2C Fig) cells. These results accorded with the finding that NBS1 phosphorylation was critical to MRN stimulation [9, 34, 35, 39]. Unlike the NDV group, the UV-NDV group did not activate the phosphorylation of endogenous NBS1 (Fig 7B), indicating that NDV lytic replication was required to activate the MRN complex. To further confirm the role of NBS1 activation in response to NDV replication and membrane fusion, we subsequently detected the spatial location of phosphorylated NBS1 (Ser 343) in A549 cells through IFA. As expected, we observed that the aggregation of bright punctum foci of phosphorylated NBS1 in the nuclei and cytoplasm in the NDV (Fig 7D, vertical lines 1 and 2) and the F and HN co-expression (Fig 7D, vertical lines 3 to 4) groups, in contrast to the negative control cells with phosphorylated NBS1 were broadly distributed in the cytoplasm without forming bright foci in the nuclei (Fig 7D, vertical line 7). This result was consistent with the Western blot results (Fig 7A to 7C). Interestingly, immunoblotting analysis indicated a slightly different effect on endogenous NBS1 expression between the virulent NDV infection (Fig 7A, horizontal line 4) and F-HN co-expression (Fig 7C, horizontal line 4) groups, suggesting that the role of NBS1 in ATM-mediated DSB may differ in response to NDV infection and membrane fusion. To confirm this result, we subsequently observed the spatial redistribution of endogenous NBS1 in A549 and NCI-H1975 cells by IFA. As expected, significant aggregations of endogenous NBS1 in nuclei were observed in both the NDV-infection and the F-HN co-expression groups in A549 (S7A Fig) and NCI-1975 (S7B Fig) cells, consistent with the results after ionizing irradiation [34, 40]. Considering that the NBS1 protein itself, as an ATM substrate, seemed to serve as an adaptor in the phosphorylation of other ATM substrates [9], we speculated that all these changes in endogenous NBS1 were rapid processes of signal transduction to recruit certain downstream ATM substrates.

To further evaluate the activation state of the MRN complex, we performed Western blot analysis to detect the total level of Mre11 and RAD50 in A549, NCI-1299, and NCI-1975 cells in response to NDV infection and F-HN co-transfection. The immunoblotting results of Mre11 suggested slightly up-regulation in A549 (Fig 7A, horizontal line 1 and S1C Fig), NCI-H1975 (S2A Fig), and NCI-1299 (S2B Fig) cells after NDV infection. Similarly, up-regulation of total Mre11 was observed during F-HN co-expression in A549 (Fig 7C), NCI-H1975 (S2A and S2C Fig), and NCI-H1299 (S2B and S2C Fig) cells. Meanwhile, the expression of endogenous RAD50 was up-regulated during late NDV infection or F-HN co-transfected in A549 (Fig 7A and 7C, horizontal line 2 and S1C Fig), NCI-H1975 (S2A and S2C Fig), and NCI-H1299 (S2B and S2C Fig) cells. Considering that Mre11, RAD50, and NBS1 form foci at sites of DNA damage in response to DSBs [37, 40, 41], we subsequently examined the spatial distribution of MRN complexes in A549, NCI-H1975, and NCI-H1299 cells. As expected, many aggregated nuclear foci of Mre11 (S7C and S7D Fig) and RAD 50 (S8 Fig) were observed in A549, NCI-H1299 and NCI-1975 cells in response to both NDV infection and F-HN co-expression, indicating that late NDV infection and syncytium formation activated and altered the reconstitution of the MRN complex. This complex was recruited to the DNA DSB site and was involved in the induction of the ATM-dependent signaling cascade. Collectively, these results combined with those from previous studies [2, 14, 37] indicated that the MRN complex played roles as upstream sensor of the ATM-mediated DSB signal.

### MRN complex activity was essential for NDV replication and membrane fusion in tumor cells

**Fig 8:**
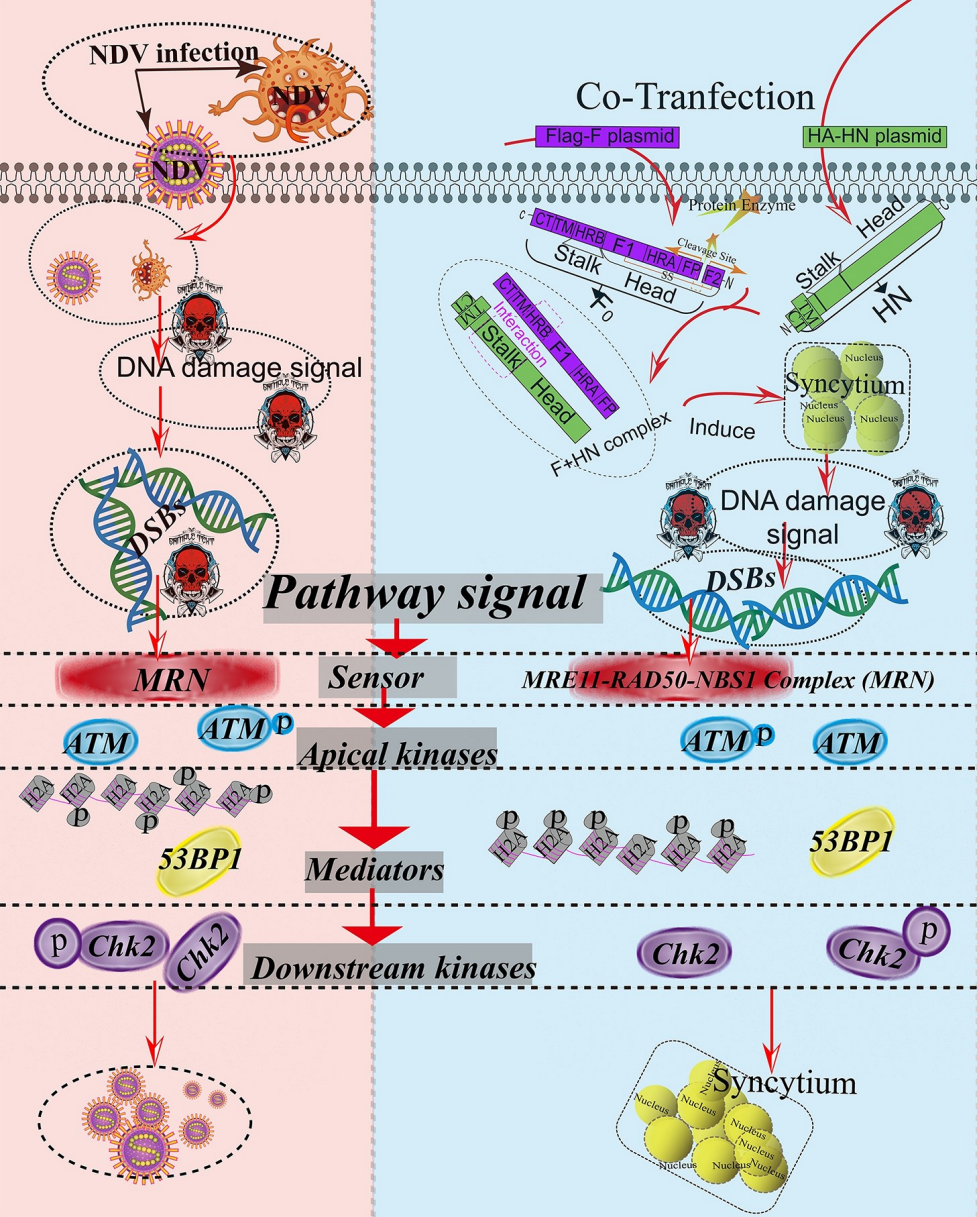
Schematic of the ATM-mediated DNA double-strand breaks (DSBs) triggered by virulent NDV infection and membrane fusion. Virulent oncolytic NDV infects target tumor cells through viral HN-mediated attachment. It then triggers F protein conformational changes and releases fusion peptides to fuse the viral and cellular membranes [29, 30]. Meanwhile, after transfection, the inactive precursor of F protein (F_0_) is proteolytically cleaved by the host-cell protease at the cleavage site and forms a biologically active protein comprising the disulfide-linked F_1_+F_2_ heterodimer [29–31]. After a series of proteolytic processing and conformational changes, the fusogenically active F_1_ protein and HN form an F+HN complex via the interaction between a stalk and an HRB domain [29, 30]. Once triggered, HRB completely refolds around HRA, thereby forming stable 6 HB and a fusion pore [30] and consequently inducing membrane fusion and syncytium formation. The virulent oncolytic NDV infection and F-HN co-expression induce DSBs in tumor cells, leading to the recruitment of the MRN complex and the separation of the dimeric, inactive form of ATM into a monomeric, phosphorylated form, which is further activated by the MRN complex through directly binding ATM at DSB sites [10]. ATM activation by DSBs occurs in two steps [2, 14]. Activated ATM initially triggers a rapid cascade of phosphorylation events involving H2AX, 53BP1, and Chk2. Mediators and signal transducers are then recruited to amplify the signal, which finally reaches effector proteins that trigger the cellular response to DNA damage [1, 6, 18]. Eventually, ATM-mediated DSBs facilitate oncolytic NDV replication and promote syncytium formation. The solid arrows indicate the stimulation and protein requirements for accumulation.

We next investigated the effects of the MRN complex on NDV replication and membrane fusion. MRN kinase activity was blocked using Mirin [42], a specific pharmacological inhibitor of the MRN complex. The Mirin inhibition of MRN activity was evaluated based on NBS1 phosphorylation, as phosphorylated NBS1 on Ser343 is a specific phosphorylation site in response to ATM-mediated DSB [14, 39, 43–45]. Compared with the mock control, Mirin treatment significantly inhibited the NDV infection-dependent phosphorylation of NBS1 in a time- and dose-dependent manner (Fig 7E, vertical lines 5 and 9, and Fig 7F, vertical lines 7 to 9), suggesting that the specific inhibitor of MRN was effective. Compared with the DMSO/NDV group, the Mirin treatment group (NDV+Mirin) successfully blocked the NDV infection-dependent ATM-mediated DSB downstream signal, as evinced by the phosphorylation of ATM, Chk2, and H2A.X (Fig 7E and 7F, horizontal lines 3, 5, and 7), suggesting the requirement for MRN in ATM activation. Compared with the DMSO/NDV group, Mirin treatment significantly inhibited intracellular progeny virion replication indicated by the NDV NP and prevented extracellular progeny production measured by TCID_50_ in a time-dependent (Fig 7E and 7G) and dose-dependent (Fig 7F and 7H) manners. Similarly, the drug-treated group (Mirin+F-HN) significantly blocked the membrane fusion and syncytium formation compared with the F-HN group. This phenomenon was proven by the statistical analysis of the syncytium number (Fig 7I) and Western blot analysis (Fig 7J). These results suggested that MRN complex activity was required for membrane fusion and oncolytic NDV replication in tumor cells (Fig 8). In summary, the activation of MRN was essential for sensing and signaling ATM-mediated DSBs in tumor cells, which, in turn, was necessary for membrane fusion and oncolytic NDV replication in tumor cells (Fig 8).

## Discussion

Most viruses have evolved a plethora of strategies to activate DNA damage machine as they commandeer cells to maximize their own replication [1, 6, 19–21]. Several viruses, such as wild-type adenovirus [46] and Herpes simplex virus type 1 (HSV-1) [47, 48], also possess mechanisms to inactivate the downstream three-tied DDR signal. Most previous work has focused on the relationship between DNA viruses, especially oncogenic DNA viruses [18], and the host DDR pathway. Few studies, however, have reported on the relationship between the DDR pathway and RNA viruses [21], especially oncolytic NDV, a single-strand, negative-sense RNA virus. In the present study, we clearly identified that NDV infection and F-HN co-expression activated upstream ATM and ATR kinases, as characterized by the indicator activation of ATM (Fig 2, S1 to S5 Figs), and ATR (S9 Fig). Considering that ATM-kinase related DSB responses are among the most deleterious forms of DNA chromosome breakage [7, 9], we focused on the relationship among virulent NDV infection, membrane fusion, and ATM-mediated DSBs.

A regulatory protein network involved in ATM-mediated DSBs, including MRN complex sensors, mediators, downstream kinases, and effectors, is known to change their localization in response to various DNA damage stresses [17]. In the present study, we proved that NDV infection and membrane fusion activated ATM-mediated DSB response, as evinced by ATM auto-phosphorylation on Ser1981 accompanied by its monomerization and the phosphorylation of downstream substrate markers. The redistribution of the distinct spatiotemporal dynamics of the pan-nuclear punctate foci of phosphorylated ATM (S4 and S5 Figs), H2A.X (Fig 3 and S6A Fig), 53BP1 (Fig 4 and S6B Fig), and Chk2 (Fig 5 and S6C Fig) was observed in response to NDV infection and F-HN co-expression, confirming the activation of ATM-mediated DSBs. We also observed the formation of γ-H2A.X nucleus punctum foci during NDV infection and membrane fusion, supporting the role of γ-H2A.X as a signal for recruiting DNA damage proteins to DSB sites in an ATM-dependent manner [10, 41]. Interesting, histone H2A.X also participates in the surveillance of replicational stress in an ATR-dependent manner, indicating the multiple roles of H2A.X in DDR [49]. In the present study, we confirmed that the phosphorylation and nucleus aggregation of H2A.X was activated in an ATM-dependent manner (Figs 2 and 3, S1 to S3 and S6 Figs) rather than in an ATR-dependent manner (S9 Fig). The protein 53BP1, originally identified as p53-interacting protein, is also now viewed as a candidate ATM coactivator that colocalizes with γ-H2A.X to form nuclear foci in response to DSBs [50]. Accordingly, we inferred that these pan-nuclear punctum foci of 53BP1 may serve as powerful platforms to amplify DSBs via repeated cycles of γ-H2A.X, which is consistent with the requirement of 53BP1 recruitment during HSV-1 infection [48]. The specific ATM-dependent Chk2 phosphorylation on Thr 68, which is required for Chk2 activation, was completely restricted to DSB sites [34]. Given that Chk2 requires direct or indirect contact with DSB sites before it can spread throughout nuclei as an active kinase [34], we inferred that Chk2 was a candidate ATM-Chk2 axis transmitter [3], enabling coordinated pan-nuclear aggregation to DNA damage sites. Although we observed that many components of the multifaceted ATM markedly underwent spatial redistribution in response to DSB signal activation triggered by virulent NDV infection and membrane fusion, the significance of the abovementioned nuclear aggregation foci in nuclei and the exact focal-assembly mechanism of these damage-stress foci remain unclear.

We also found that KU55933, a specific pharmacological inhibitor of ATM activation [36], suppressed intracellular and extracellular NDV replication in a time- and dose-dependent manner (Fig 6A to 6F). This finding was consistent with the replication characteristic of the minute virus of canines [51] and hepatitis C virus [52], suggesting that ATM kinase was required for NDV replication. Expectedly, KU55933 also significantly blocked membrane fusion (Fig 6G and 6H), indicating that syncytium formation required activated ATM kinase. We further found that F cleavage ability functioned on the activation of ATM-mediated DSB signals (Fig 2D and S2C Fig), extending our prior understanding of membrane fusion. Overall, this work provided the first evidence of oncolytic NDV infection and membrane-fusion manipulation of ATM-dependent DSB signal activation to benefit virus replication and promote syncytia formation in tumor cells (Fig 8). We hypothesized several possible mechanisms underlying late NDV infection and syncytium formation harnessing ATM-mediated DSBs or circumventing them to prevent undesirable downstream consequences. The recruitment of DNA repair factors to DNA replication centers is a common theme among DNA viruses, as they proofread and resolve their genomes prior to packaging [18] (except HSV-1 [48]). For example, ATM and ATR contribute to Simian virus 40 robust replication by directly promoting the repair of replication-associated DSBs and alleviating the breakage of converging forks, respectively [53]. Although the life cycles of DNA and RNA viruses largely differ, we speculated that activated ATM (Fig 2, S1 to S5 Figs), and ATR (S9 Fig) may play similar roles in tumor cells in response to NDV infection and membrane fusion, as evinced by the activation of DNA-PKs signals, regulators of the nonhomologous end joining-mediated repair of DSBs. We hypothesized that the nuclear aggregation of a series of ATM substrates may facilitate signal-cascade transduction at DSB sites, especially DNA repair factors, such as DNA-PKs and the Ku70-Ku80 heterodimer. Additionally, the ATM and ATR pathways indirectly regulate cell-cycle progression and apoptosis through the phosphorylation of a series of downstream checkpoint kinases, such as 53BP1, p53, and Chk1/2 [18]. Considering the phosphorylation of downstream ATM targets, including H2A.X (Fig 3 and S6 Fig), 53BP1 (Fig 4 and S6 Fig), Chk1 (S9 Fig), and Chk2 (Fig 5 and S6 Fig), we accordingly proposed that ATM may mediate cell-cycle checkpoints and apoptosis and eventually facilitate replication viral progeny replication and membrane fusion.

The MRN complex possesses nuclease- and DNA-binding properties, which specifically senses DSB signals and recruit ATM kinase to the ends of DSBs [2, 7, 54]. Adenovirus infection blocks the ATM/ATR-mediated DDR through oncoprotein reorganization (E1b55K/E4orf6 and Edorf3) and degradation of the MRN complex [46]. In the present study, based on the results of Western blot analysis and IFA (Fig 7 and S1 to S3 Figs and S7 and S8 Figs), we demonstrated that NDV infection and membrane fusion activated MRN complex activity. Significant nuclear aggregation of NBS1, *p*-NBS1, Mre11, and RAD50 in response to NDV infection and F-HN co-expression was observed. This finding was consistent with MRN complexes inducing the rapid formation of prominent foci at damage sites [37, 40]. Given that MRN is a sensor required for ATM-mediated DSB activation [2, 14, 39], we speculated that the conformational change of endogenous MRN complexes increased their affinity for the activation of ATM-dependent downstream targets [35], such as H2A.X, 53BP1, Chk1/2, and p53. Notably, the timepoints of MRN complex activation differed between NDV infection (about 8 h.p.i.) and F-HN co-expression (about 18 h.p.t.), as indicated by the phosphorylation of NBS1 (Fig 7A and 7C, horizontal line 3). Compared with the smart NDV infection process, the process of syncytium formation mechanically triggered by F-HN interaction was slightly monotonous and tedious. Apparently, F-HN took some time to mechanically trigger sufficient numbers of syncytia and then activate of MRN sensors (Fig 7C) and ATM-mediated DSBs (Fig 2D). Meanwhile, the activation timepoint of the MRN complex sensor (about 8 h.p.i.) (Fig 7A) was slight earlier than that of ATM kinase (about 9 h.p.t.) (Fig 2A) in response to NDV infection, indicating that the activation of MRN complexes occurred slightly earlier during the transduction of the DSB signals than that of ATM kinase. Combined with a series of previous studies [2, 14, 37], we viewed that the MRN complex played a role upstream as a sensor and subsequently activated ATM-mediated DSBs. Based on the results of the Mirin drug experiment (Fig 7), a specific inhibitor of MRN [42], we identified that MRN activity was essential for virulent replication and syncytium formation. However, the multiple-activation mechanism requires further elucidation. Here, we speculated that the ATM activated by virulent NDV infection and membrane fusion followed the two-step ATM activation model [14]: First, the MRN-associated DNA tethering component was activated and then the MRN activation led to ATM recruitment and auto-phosphorylation (Fig 8).

Here, we provide several possible explanations why late NDV infection significantly provoked the ATM-dependent DSB signaling pathway in tumor cells to facilitate progeny replication. First, UV-NDV infection cannot activate ATM-mediated DSBs (Fig 2B), suggesting that ATM-mediated DDR was activated by alive NDV replication. Based on the extracellular and intracellular viral titer data (Fig 2A and 2B), we speculated that sufficient number of virus particles and replication stresses may as one of inducible factors for the initiation of DDR signaling. Second, considering that DDR can activate the interferon signal [55], we speculated that early NDV infection did not activate the ATM-dependent DSBs as a survival tactic to escape the host’s intrinsic immune-response repression. Conversely, extensive NDV propagation may generate aberrant DNA structures and all kinds of chromatin damage marks that provoked the cellular ATM-mediated DSBs. Third, unlike DNA viruses, which can directly provoke cellular DDR [18], we proposed that late NDV infection indirectly activated ATM-mediated DSBs possibly depending on cellular genotoxic or metabolic by-products, including DNA strand cross-links and oxidative DNA adducts. Fourth, considering that specific viral proteins can activate DDR signals (e.g., E1 of papillomavirus [56]) or inactivate them (e.g., ICP0 of HSV-1 [48]), we clearly observed that F and HN of virulent NDV, rather than other structural or non-structural viral proteins (Fig 2 and S2 and S3 Figs), acted synergistically to trigger the DDR pathway. Undoubtedly, the elaborate underlying mechanism remains to be elucidated in further research. We hypothesized that viral glycoprotein activated the ATM-mediated DSBs through aberrant DNA structures either directly by interacting with cellular fusogens [57, 58] or indirectly by modulating cell-cycle progression via the promotion of inappropriate S phase entry [18]. Finally, in addition to the direct effects of viral replication stress or viral proteins on the DDR signaling pathway, numerous examples of indirect effects are available on genomic integrity and DDR, such as post-translational modification (PTM) and the induction of reactive species. PTM is critical to cellular DNA repair machinery by affecting localization, protein-protein interactions, and protein activity [1]. Histones are subject to a plethora of complex and dynamic PTMs, including typical phosphorylation, acetylation, SUMOylation, ubiquitination, methylation, and epigenetic reprogramming, which all define the degree of DNA accessibility and facilitate protein recruitment [1, 59]. Phosphorylation modification is considered a central signaling marker in cellular DDR [1]. In this study, oncolytic NDV infection significantly up-regulated the phosphorylation of ATM, H2A.X, and Chk2 in tumor cells, suggesting that NDV infection indeed altered the phosphorylation of PTM signals. Meanwhile, the cellular ubiquitin conjugation system is emerging as an important aspect of protecting genome integrity [59]. Two RING-type ubiquitin E3 ligases, RNF8 and RNF168, participate in the early signaling of DSBs, as their ubiquitination activity is required for the further recruitment of downstream regulators and repair factors for damaged chromatin, such as 53BP1, RAP80, and the BRCA1 complex [59–61]. The possible involvement of RNF168 and/or RNF8-mediated ubiquitylation in 53BP1 focal formation induced by NDV infection, such as HSV-1 [48], requires further elucidation. Moreover, the SUMOylation of small ubiquitin-related modifier accumulation at DSB sites to promote DSBs is also important [62]. Several histone acetyltransferases recruited to acetylate ATM complex, such as acetylase Tip60 [63] and hMORF [64], are also cofactors for ATM activation in vivo [7]. All these previous findings reveal that cells activate a series of PTMs to leave marks that can influence subsequent cellular DDR activation. We speculated that the ATM-mediated DSBs induced by oncolytic NDV infection utilized acetylation-, SUMOylation-, and ubiquitination-dependent protein-protein interactions to coordinate the amplification of DDR signals. Therefore, the cross-talk between PTMs in DSBs during NDV infection remains to be elucidated.

In summary, we identified for the first time that the co-expression of F and HN and oncolytic NDV infection activated the ATM-mediated DSB signaling pathway in tumor cells via the ATM-Chk2 axis, which then promoted syncytium formation and facilitated NDV replication. Our findings indicated that oncolytic the NDV can activate host DNA damage machinery for its own replication. These results further extend prior understanding of the membrane-fusion process and NDV infection and support the notion that the activation of the DDR pathway is not just limited to DNA virus infection. These findings reveal new links among oncolytic NDV replication, membrane fusion, and DDR as well as deepen therapeutic insights into NDV oncolytic characteristics in tumor cells.

## Materials and methods

### Cell culture, pharmacological reagents, and antibodies

Chicken embryo fibroblast (DF-1), human embryonic kidney 293 T (HEK 293T), and adenocarcinomic human alveolar basal epithelial (A549) cell lines were purchased from the American Type Culture Collection (Manassas, VA, USA). DF-1 and HEK 293T were cultured in Dulbecco’s Modified Eagle Medium (DMEM) supplemented with 10% fetal bovine serum (FBS) (Thermo Fisher Scientific, Waltham, MA, USA). A549 cells were cultured in DMEM nutrient mixture F-12 (DMEM-/F-12) supplemented with 10% FBS. NCI-H1975 and NCI-H1299 cell lines were purchased from the Cell Bank of the Shanghai Institute of Biochemistry and Cell Biology, Chinese Academy of Science (http://www.cellbank.org.cn) and were cultured in RPMI-1640 medium supplemented with 10% FBS.

Phalloidin (P2495), a labeled fluorescein isothiocyanate, was used to identify the polymeric and oligomeric forms of F-actin filaments instead of monomeric actin (Sigma Aldrich, St. Louis, MO, USA). Stock solutions of phalloidin conjugates were prepared in dimethyl sulfoxide (DMSO) at 0.5 mg/ml. Etoposide (S1225) was used as the DNA damage inducer, KU-55933(S1092) was the ATM kinase specific inhibitor, Mirin (S8096) was the MRN kinase inhibitor, and VE-821 (S8007) was the ATR kinase specific inhibitor. All these drug reagents were purchased from Selleck Chemicals Company (Houston, TX, USA), diluted, and used according to their specifications. All reagents were stored at −20°C and diluted to the desired working concentration in fresh medium immediately before use.

Primary antibodies including mouse anti-NP monoclonal antibody were prepared in our laboratory. Anti-rabbit-Flag (7425) and Anti-mouse-Flag (1804) were purchased from Sigma Aldrich (St. Louis, MO, USA). Anti-β-actin (3700), anti-H2A (7631T), anti-HA (C29F4), anti-Chk2 (6334T), anti-53BP1(4937S), anti-ATM (2873), anti-PARP (9532), anti-cleaved PARP (5625), anti-phospho H2AX (Ser 139) (9718), anti-phospho Chk2 (Thr 68) (2197T), MRN antibody sample kit (8344) comprising anti-phospho-Nbs1 (Ser 343) (3001), Mre1 (4847), Nbs1 (14956), and anti-phospho-ATR (Ser428) (2853T), and anti-phospho-Chk1 (S345) (2348T) were purchased from Cell Signaling Technology (Beverly, MA, USA). Anti-RAD50 (ab124682), anti-ATR (Ser 428) (ab2905), anti-Chk1 (ab40866), and anti-phospho-ATM (Ser 1981) (ab81292) were purchased from Abcam (Cambridge, MA, USA). Secondary antibodies, including horseradish peroxidase-conjugated (HRP) goat anti-rabbit or goat anti-mouse IgG antibodies (Merck Millipore, Billerica, MA, USA) were validated for use in Western blot analysis. The goat anti-mouse and goat anti-rabbit Alexa Fluor secondary antibodies, including Alexa Fluor 488 and 594 (molecular probes) used for indirect immunofluorescence, were purchased from Thermo Fisher Scientific (Waltham, MA, USA).

### Plasmid construction, virus, and UV inactivation of NDV

Flag–F and HA–HN plasmids were provided by Prof. Sa Xiao (Northwest A&F University, Shaanxi, China). Both viral protein plasmids were constructed based on the F48E9 strain sequence (GenBank accession number MG456905), a standard velogenic strain. Seven F protein mutation plasmids were successfully constructed following the manufacturer’s instruction for the Mut Express Multis Fast Mutagenesis Kit V2 (Vazyme, Nanjing, China) [31]. The pCAGGS-F49E9-N-myc-L (abbreviated as “pCAGGS-myc-L”) plasmid was also constructed based on the sequence of F48E9 strain with the following primers:

pCAGGS-F48-myc-L-forward (EcoRI site underlined): AAGAGGATCTGGAATTCATGGCGAGCTCCGGTCCCGAGAGGGC;

pCAGGS-F48-myc-L-reverse (KpnI site underlined): GGCATGCCCGGGTACCAGAGTCACAGTTACTGTAATATCCCTTG.

In this study, the Flag-M and Flag-M^ΔNLS(247aa~263aa)^ plasmids that deleted the NLS were provided by Dr. Weiyu Jing. Flag-NP, Flag-P, Flag-V, and Flag-W plasmids were obtained from Dr. Yingjie Sun. All six viral plasmids were constructed based on the NDV Herts33 strain, a standard velogenic NDV strain. According to OIE standard criteria, F48E9 and Herts/33 strains are velogenic strains and have the same pathogenicity characteristic. Additionally, pCAGGS-La Sota F-C-Flag and pCAGGS-La Sota-N-HA-HN (abbreviated as “La-Flag-F” and “La-HA-HN”, respectively) were constructed based on the NDV La Sota strain (GenBank accession number MG456905), a standard lentogenic vaccine strain, with the following primers:

pCAGGS-LaSota-F-Flag-C for (EcoRI site underlined): AAGAGGATCTGGAATTCATGGGCTCCAGACCTTCTACCAAG;

pCAGGS-LaSota-F-Flag-C Rev (KpnI site underlined): GGCATGCCCGGGTACCTTTATCGTCATCGTCTTTGTAGTCCATTTTTGTAGTGGCTCTCATCTG;

pCAGGS-LaSota-N-HA-HN-for (EcoRI site underlined): GTTCCAGATTACGCTGAATTCATGGACCGCGCCGTTAGCCAAGTTG; pCAGGS-LaSota-N-HA-HN-Rev (KpnI site underlined): AGGCATGCCCGGGTACCGCCAGACCTGGCTTCTCTAACCCCG.

All these plasmids were confirmed by sequencing.

NDV strain Herts/33 and La Sota were obtained from the China Institute of Veterinary Drug Control (Beijing, China). Allantoic fluid (3 ml) of Herts/33 inoculation was exposed to UV at 75 mW/cm^2^ using a low-pressure mercury vapor discharge lamp to obtain the replication-incomplete Herts/33 NDV. After UV exposure, we confirmed the NDV infectivity by the lack of replication in 9-day-old specific pathogen-free embryonated chicken eggs.

### Transfection, viral infection, and drug treatment or UV exposure

For experimental transfections, the cells were cultured at approximately 70–80% confluence in six-well plates and transfected with 1–2μg each of expression plasmids (Flag–F and HA–HN) by using a Lipofectamine 2000 Transfection Reagent (Thermo Fisher Scientific, Waltham, MA, USA) or FuGEN HD (Promega, Madison, WI, USA) in accordance with the manufacturer’s guidelines. In a typical procedure, the calculated plasmid DNA solution was mixed with a transfection reagent in an Opti-MEM reduced-serum medium for 15 min at room temperature and the mixture was added dropwise to the cells. The cells were incubated at 37°C with 0.25% CO_2_ for 4 h, washed twice with Opti-MEM medium, and cultured in DMEM-/F-12 or RPMI-1640 medium supplemented with 10% FBS for further experiments.

For NDV infection, DF-1, A549, NCI-H1975, and NCI-H1299 cells were infected with NDV at a multiplicity of infection (MOI) equal to 1 in a 37°C cell-culture incubator. Following absorption for 1 h, the unattached viruses were removed, and the cells were washed three times with phosphate-buffered saline (PBS) and cultured in complete medium. After viral infection, the cell samples were used for Western blot analysis and IFA.

For pharmacological experiments, we first determined the experimental drug concentrations by WST-1 assay according to the manufacturer’s guidelines. A549, NCI-H1975, and NCI-H1299 cells were treated with etoposide and cultured with complete DMEM-/F-12 supplemented with 10% FBS in a 37°C cell culture incubator at an indicated timepoint. The cell samples were collected for Western blot analysis and IFA. Etoposide treatment induced the formation of DSBs by trapping topoisomerase II-DNA covalent complexes [6]. DNA damage was produced by UV radiation and etoposide treatment. The main factor to consider when taking the positive control for IFA examination was the marker protein expression of DSB signals to UV or etoposide. A549 cells were pretreated with a working concentration of KU55933, Mirin, or VE821 for 1 h prior to NDV infection. Viral infection was conducted as described above. Extracellular NDV titers were determined on DF-1 cells as the median tissue culture infective dose (TCID_50_) based on the classical Reed–Muench method. Intracellular NDV titers were determined as the expression of NDV NP protein with monoclonal antibody against NDV NP protein by Western blot analysis. For the UV exposure experiment, A549, NCI-H1975, and NCI-H1299 cells were irradiated with UV for corresponding minutes as indicated at 75 mW/cm^2^ using a low-pressure mercury vapor discharge lamp. Afterwards, the cell samples were used for Western blot analysis and IFA.

### Western blot analysis

Cell samples were washed with PBS and lysed with lysis buffer (2% sodium dodecylsulfate [SDS], 10% glycerin, 5% 2-mercaptoethanol and 0.1% bromophenol blue). The lysates were collected into 1.5 ml tubes on ice for 30 min and centrifuged at 12, 000 revolutions per minute (rpm) at 4°C for 15 min for clarification. The lysates were diluted into 5× SDS buffer to a 1× SDS final concentration. Denaturation was performed at 100°C for 10 min. An equal amount of these prepared samples was subjected to SDS–polyacrylamide gel electrophoresis and transferred onto a nitrocellulose blotting membrane (GE Healthcare Life Science, Amersham, Protran 0.2 or 0.45 NC, Germany). The membranes were then blocked with 5% skimmed milk in 0.05% Tris-buffered saline containing 0.05% Tween 20 (TBST) for 30 min at room temperature. The membranes were washed thrice with 0.05% TBST (5 min/each) and inoculated with primary antibodies for at least 6 h at 4°C. The membranes were washed thrice again with 0.05% TBST (5 min/each), and incubated with HRP-conjugated secondary antibodies at room temperature for 2 h. Lastly, after washing thrice again, the antibody–antigen complex was exposed with a chemiluminescence reagent solution kit (Share-bio Biotechnology, Shanghai, China) using a multi-chemiluminescence image analysis system (Tanon 5200, Tanon, Guangzhou, China).

### Indirect immunofluorescence assay (IFA)

The A549, NCI-H1975, and NCI-H1299 cells were grown on coverslips in a six-well plate and transfected. After transfection, the coverslips were washed with PBS and fixed with 4% paraformaldehyde for 30 min at room temperature. The cells were permeabilized with 0.5% Triton X-100 for 10 min at room temperature, washed thrice with PBS, blocked with 3% bovine serum albumin at 37°C for 30 min, inoculated with a primary antibody for 2 h, and washed three times again with PBS. Antibody binding was detected using secondary antibodies conjugated with Alexa Fluor 488, 594, or 633 for 1 h in a moist container in the dark at 37°C. The cells were then stained with DAPI (0.1 μg/ml) for 8 min at 37°C. Finally, the coverslips were mounted on the microslides and air dried. Fluorescent images were visualized and captured on a confocal fluorescence microscope (ZEISS LSM880, Germany).

The procedure for phalloidin-labeled F-actin was as follows. The prepared cell samples were fixed with 4% formaldehyde solution and permeabilized with 0.1% Triton X-100. The cells were then stained at a final concentration of 50 μg/ml fluorescent phalloidin conjugate solution in PBS for 40 min at room temperature and finally stained with DAPI after washing thrice with PBS.

### Statistical analyses

Statistical analyses were performed using GraphPad Prism software (Graph Pad Software, Inc., San Diego, CA, USA). All values are representative or the mean of at least three independent experiments. Standard deviation and standard error of the mean were calculated and determined using at least three technical replicates (unless stated otherwise). Three or more means from the time-course study were analyzed by Student’s *t* analysis of variance (ANOVA). Statistical significance was evaluated by determining *p*-values through the two-tailed Student’s t-test (two-tailed distribution). ns, *p* > 0.05; *p < 0.05; **, *p* < 0.01, ***, *p* < 0.001.

## Supporting information

S1 Fig Ultraviolet exposure, etoposide treatment, and velogenic/lentogenic NDV infection activated the ATM-mediated DSB signaling. **(A)** Ultraviolet (UV) exposure and etoposide treatment activated ATM-mediated DSB signaling in A549 cells. Cells treated with UV and etoposide were used as positive controls for inducing DDR. Western blot samples were prepared from A549 cells after UV exposure at 75 mW/cm^2^ using a low-pressure mercury vapor discharge lamp corresponding to the marked timepoints (15, 30, 45, and 60 min). A549 cells were treated with etoposide at working concentrations of 10, 20, 40, 50, and 80 μm for 24 h. After UV exposure and etoposide treatments, we then conducted Western blot analysis in accordance with the procedures in the Materials and Methods section. The monomer ATM was marked with a black triangle. β-actin served as a loading control. **(B)** UV and etoposide treatments activated the MRN sensor of ATM-mediated DSB signaling in A549 cells. Cells treated with UV and etoposide served as positive controls for DDR induction. **(C)** Velogenic NDV and lentogenic NDV strains activated the ATM-mediated DSB signaling in A549 cells. Western blot samples were prepared from A549 cells with lentogenic NDV infection (La Sota strain, MOI = 1 or 10) or virulent NDV (Herts/33 strain, MOI = 1) corresponding to the marked timepoints and analyzed in accordance with the procedures in the Materials and Methods section.
(TIF)

S2 Fig Virulent NDV infection and membrane fusion activated ATM-mediated DSBs and MRN complex signals in A549, NCI-H1975, and NCI-H1299 cells. **(A)** Virulent NDV infection and membrane fusion activated ATM-mediated DSB signals and MRN complex signals in NCI-H1975 cells as discovered by Western blot analysis. Samples were prepared from NCI-H1975 cells after virulent oncolytic NDV infection (Herts/33 strain, MOI = 1) corresponding to the marked timepoints, UV-exposed for 45 min, and treated with etoposide at a final concentration of 80 μm for 24 h, and then co-transfected with both Flag-F and HA-HN plasmids for 24 h and 48 h. Cells treated with UV and etoposide were used as a positive controls for DDR induction. The monomer ATM was marked with a black triangle. **(B)** Virulent NDV infection and membrane fusion activated ATM-mediated DSB signals and MRN complex signals in NCI-H1299 cells as discovered by Western blot analysis. Samples were prepared from NCI-H1299 cells after virulent oncolytic NDV infection (Herts/33 strain, MOI = 1) corresponding to the marked timepoints, UV-exposed for 45 min, and treated with etoposide at a final concentration of 80 μm for 24 h, and then co-transfected with both Flag-F and HA-HN plasmids for 24 h and 36 h. **(C)** Membrane fusion triggered by F and HN of velogenic NDV activated ATM-mediated DSBs signal in A549, NCI-H1975, and NCI-H1299 cells as discovered by Western blot analysis. A549, NCI-H1975, and NCI-1299 cells were mock-transfected or co-transfected with both La-Flag-F and La-HA-HN plasmids or both Flag-F and HA-HN plasmids for 36 h.
(TIF)

S3 Fig F and HN of virulent NDV cooperated synergistically to activate ATM-mediated DSB signaling. **(A)** Subcellular localization of structural and non-structural protein of virulent oncolytic NDV in A549 cells. A549 cells were transfected with Flag-NP, Flag-P, Flag-M, Flag-M^ΔNLS^, pCAGGS-Myc-L, Flag-V, Flag-W, HA-HN, Flag-F, and F-HN for 24 h in A549 cells. Flag-Tag (Red); nuclei (blue); HA-Tag (Green). Scale bars = 20 μm. **(B)** Synergistic cooperation of F and HN activated ATM-dependent DSBs as discovered by Western blot analysis. A549 cells were mock-transfected or transfected with Flag-NP, Flag-P, Flag-M, Flag-M^ΔNLS^, pCAGGS-Myc-L, Flag-V, Flag-W, HA-HN, Flag-F, and F-HN for 36 h. After transfection, we then conducted Western blot analyzed in accordance with the procedures in the Materials and Methods section. The monomer ATM was marked with a black triangle. **(C)** The structural M protein of NDV did not activate the ATM-mediated DSBs pathway in A549 cells. A549 cells were transfected with Flag-M and Flag-M^ΔNLS^ for 36 h. *p*-ATM (Ser 1981), γ-H2A.X (Ser 139), and *p*-Chk2 (Thr 48) were stained with green, nuclei with blue, Flag-tag with green. The red arrow indicates the transfected cells and black arrow indicates mock-transfected cells used as a itself control.
(TIF)

S4 Fig Virulent NDV infection and membrane fusion altered the subcellular localization of ATM phosphorylation on Ser1981 in A549 cells. **(A)** Subcellular localization of ATM phosphorylation on Ser1981 in response to virulent oncolytic NDV infection in A549 cells. A549 cells were mock-infected and NDV-infected (Herts/33, MOI = 1) for 18 h and 30 h, UV exposed for 30 min, and treated with etoposide at a final concentration of 80 μm for 24 h. Phosphorylated ATM (green); nuclei (blue); NDV (red). The arrow indicates localization of phosphorylated ATM in cytoplasm. Scale bars = 20 μm. **(B)** Subcellular localization of endogenous ATM phosphorylation on Ser1981 in response to F-HN co-expression in A549 cells. A549 cells were mock-transfected or co-transfected with both Flag-F and HA-HN plasmids for 24 h and 30 h.
(TIF)

S5 Fig Virulent NDV infection and membrane fusion altered the subcellular localization of ATM phosphorylation on Ser1981 in NCI-H1975 and NCI-H1299 cells. **(A)** Subcellular localization of ATM phosphorylation on Ser1981 in response to virulent oncolytic NDV infection and F-HN co-expression in NCI-H1975 cells. NCI-H1975 cells were mock-infected and NDV-infected (Herts/33, MOI = 1) for 12 h and 18 h, UV-exposed for 45 min, and treated with etoposide at a final concentration of 80 μm for 24 h. The UV and etoposide treatment groups served as the positive controls. NCI-H1975 cells were co-transfected with both Flag-F and HA-HN plasmids for 24 h. Phosphorylated ATM (green); nuclei (blue); NDV (red). The arrow indicates localization of phosphorylated ATM in cytoplasm. Scale bars = 20 μm. **(B)** Subcellular localization of ATM phosphorylation on Ser1981 in response to virulent oncolytic NDV infection and F-HN co-expression in NCI-H1299 cells. NCI-H1299 cells were mock-infected and NDV-infected (Herts/33, MOI = 1) for 18 h and 24 h, UV-exposed for 45 min, treated with etoposide at a final concentration of 80 μm for 24 h, and then co-transfected with both Flag-F and HA-HN plasmids for 24 h.
(TIF)

S6 Fig Aggregation of γ-H2A.X, 53BP-1, and phosphorylated Chk2 in the nuclei induced by virulent NDV infection and membrane fusion in NCI-H1975 cells. **(A)** Representative images showing that virulent NDV infection and F-HN co-expression triggered the nuclear aggregation of γ-H2A.X in NCI-H1975 cells. NCI-H1975 cells were mock-infected, NDV-infected (MOI = 1), inoculated for 24 h, UV-exposed for 45 min, treated with etoposide at a final concentration of 80 μm for 24 h, and then mock-transfected or co-transfected with both Flag-F and HA-HN plasmids for 24 h. The UV and etoposide treatment groups served as the positive controls. γ-H2AX (green); nuclei (blue); NDV (red). Scale bars = 20 μm. **(B)** Representative images showing that virulent NDV infection and F-HN co-expression triggered nuclear aggregation of 53BP-1 in NCI-H1975 cells. NCI-H1975 cells were mock-infected, NDV-infected (MOI = 1), inoculated for 24 h, UV-exposed for 45 min, treated with etoposide at a final concentration of 80 μm for 24 h, and then mock-transfected or co-transfected with both Flag-F and HA-HN plasmids for 24 h. 53BP1 (green); nuclei (blue); NDV (red). **(C)** Representative images showing that virulent NDV infection and F-HN co-expression triggered nuclear aggregation of *p*-Chk2 in NCI-H1975 cells. NCI-H1975 cells were mock-infected, NDV-infected (MOI = 1), inoculated for 24 h, UV-exposed for 45 min, and treated with etoposide at a final concentration of 80 μm for 24 h, and then mock-transfected or co-transfected with both Flag-F and HA-HN plasmids for 24 h. Phosphorylated Chk2 (green); nuclei (blue); NDV (red).
(TIF)

S7 Fig Aggregation of NBS1 and Mre11 in the nuclei in response to virulent oncolytic NDV infection and F-HN co-expression in A549 and NCI-H1975 cells. **(A)** Representative images showing the spatial redistribution of NBS1 in response to virulent oncolytic NDV infection and F and HN co-expression in A549 cells. A549 cells were mock-infected or NDV-infected (MOI = 1) for 30 h, treated with etoposide at a final concentration of 80 μm for 24 h, and then mock-transfected or co-transfected with both Flag-F and HA-HN plasmids for 24 h. NBS1 (green); nuclei (blue); NDV (red). The etoposide treatment group served as the positive control. The arrow indicates the localization of NBS1 in the nucleus. Scale bars = 20 μm. **(B)** Representative images showing the spatial redistribution of NBS1 in response to virulent oncolytic NDV infection and F and HN co-expression in NCI-H1975 cells. NCI-H1975 cells were mock-infected and NDV-infected (MOI = 1) for 24 h, UV-exposed for 45 min, treated with etoposide at a final concentration of 80 μm for 24 h, and then mock-transfected or co-transfected with both Flag-F and HA-HN plasmids for 24 h. NBS1 (green); nuclei (blue); NDV (red). **(C)** Representative images showing the spatial redistribution of Mre11 in response to virulent oncolytic NDV infection and F and HN co-expression in A549 cells. A549 cells were mock-infected and NDV-infected (MOI = 1) for 30 h, treated with etoposide at a final concentration of 80 μm for 24 h, and then mock-transfected or co-transfected with both Flag-F and HA-HN plasmids for 24 h. Mre11 (green); nuclei (blue); NDV (red). **(D)** Representative images showing the spatial redistribution of Mre11 in response to virulent oncolytic NDV infection and F and HN co-expression in NCI-H1975 cells. NCI-H1975 cells were mock-infected and NDV-infected (MOI = 1) for 24 h, UV exposed for 45 min, treated with etoposide at a final concentration of 80 μm for 24 h, and then mock-transfected or co-transfected with both Flag-F and HA-HN plasmids for 24 h. Mre11 (green); nuclei (blue); NDV (red).
(TIF)

S8 Fig Aggregation of RAD50 in the nuclei in response to virulent oncolytic NDV infection and F and HN co-expression in A549, NCI-H1975, and NCI-H1299 cells. **(A)** Representative images showing the spatial redistribution of Rad50 in response to virulent oncolytic NDV infection and F and HN co-expression in A549 cells. A549 cells were mock-infected and NDV-infected (MOI = 1) for 24 h, and treated with etoposide at a final concentration of 80 μm for 24 h, and then mock-transfected or co-transfected with both Flag-F and HA-HN plasmids for 24 h. Rad50 (green); nuclei (blue); NDV (red). The etoposide treatment and UV-groups served as the positive controls. The arrow indicates localization of NBS1in the nucleus. Scale bars = 20 μm. **(B)** Representative images showing the spatial redistribution of Rad50 in response to virulent oncolytic NDV infection and F and HN co-expression in NCI-H1975 cells. NCI-H1975 cells were mock-infected and NDV-infected (MOI = 1) for 24 h, treated with etoposide at a final concentration of 80 μm for 24 h, and then mock-transfected or co-transfected with both Flag-F and HA-HN plasmids for 24 h. Rad50 (green); nuclei (blue); NDV (red). **(C)** Representative images showing the spatial redistribution of Rad50 in response to virulent oncolytic NDV infection and F and HN co-expression in NCI-H1299 cells. NCI-H1299 cells were mock-infected and NDV-infected (MOI = 1) for 24 h, and treated with etoposide at a final concentration of 80 μm for 24 h, and then mock-transfected or co-transfected with both Flag-F and HA-HN plasmids for 24 h. Rad50 (green); nuclei (blue); NDV (red).
(TIF)

S9 Fig ATR-dependent single-stranded DNA broken signaling was not required for NDV replication and membrane-fusion. **(A)** Virulent NDV infection activated ATR kinase as discovered by Western blot analysis. Samples were prepared from A549 cells after virulent oncolytic NDV infection (Herts/33 strain, MOI = 1) corresponding to the marked timepoints and analyzed in accordance with the procedures in the Materials and Methods section. β-actin served as a loading control. **(B)** UV exposure and etoposide treatment activated ATR kinase in A549 cells. Cells treated with UV and etoposide served as positive controls. Western blot samples were prepared from A549 cells after UV-exposure corresponding to the marked timepoints (15, 30, 45, and 60 min). A549 cells were treated with etoposide at working concentrations of 10, 20, 40, 50, and 80 μm for 24 h. **(C)** Representative images showing that virulent oncolytic NDV infection and F-HN co-expression triggered the nuclear aggregation of phosphorylated Chk1 on Ser345 in A549 cells. A549 cells were mock-infected or NDV-infected (MOI = 1) for 18, 24, and 30 h, UV exposed for 60 min, treated with etoposide at a final concentration of 80 μm for 24 h, and then mock-transfected or co-transfected with both Flag-F and HA-HN plasmids at the indicated timepoint. At 24 and 30 h.p.t., coverslips were examined by IFA. The UV and etoposide treatment groups served as the positive controls. *p*-Chk1 (green); nuclei (blue); NDV (red). Scale bars = 20 μm. **(D)** ATR kinase was not required for NDV intracellular replication in a dose-dependent manner. A549 cells were pretreated at a working concentration of VE-821 at 10, 20, and 30 μm for 1 h prior to NDV infection, and mock-infected, NDV-infected (MOI = 5) for 24 h. **(E)** ATR kinase was not required for NDV intracellular replication in a time-dependent manner. A549 cells were pretreated at a working concentration of VE-821 at 30 μm for 1 h prior to NDV infection, and mock-infected and NDV-infected (MOI = 5) for 18 and 24 h. **(F)** F and HN cooperated synergistically to activate ATR kinase in A549 cells. A549 cells were co-transfected with both Flag-F and HA-HN plasmids. Western blot samples corresponding to the marked timepoints were collected and analyzed in accordance with the procedures in the Materials and Methods section. **(G)** The cleavage site motif of F influenced ATR signaling activation. In A549 cells, wild-type HN plasmid were co-transfected with wild-type F, F^112G^, F^115G^, F^117L^, F^112G+115G^, F^112G+117L^, F^115G+117L^, and F^112G+115G+117L^ for 36 h. **(H)** ATR kinase activity did not influence syncytium formation in A549 cells. A549 cells were pretreated at a working concentration of VE-821 at 30 μm for 1 h prior to transfection, and co-transfected with both Flag-F and HA-HN plasmids. Syncytium formation was observed based on the number of nuclei. Numerical data were the average numbers of syncytia in different microscope fields in at least three independent experiments. Significance was analyzed using two-tailed Student’s t-test. NS, *p* > 0.05; *, *p* < 0.05; **, *p* < 0.01; ***, *p* < 0.001. Western blot samples corresponding to 18 and 36 h.p.t. were collected and analyzed.
(TIF)
