# Supplementary figures and images for: ATM-mediated DNA double-strand break response facilitated oncolytic Newcastle disease virus replication and promoted syncytium formation in tumor cells
Source: PLoS Pathog. 2020 Jun 1;16(6):e1008514. doi: 10.1371/journal.ppat.1008514 (PMC7263568; doi:10.1371/journal.ppat.1008514)

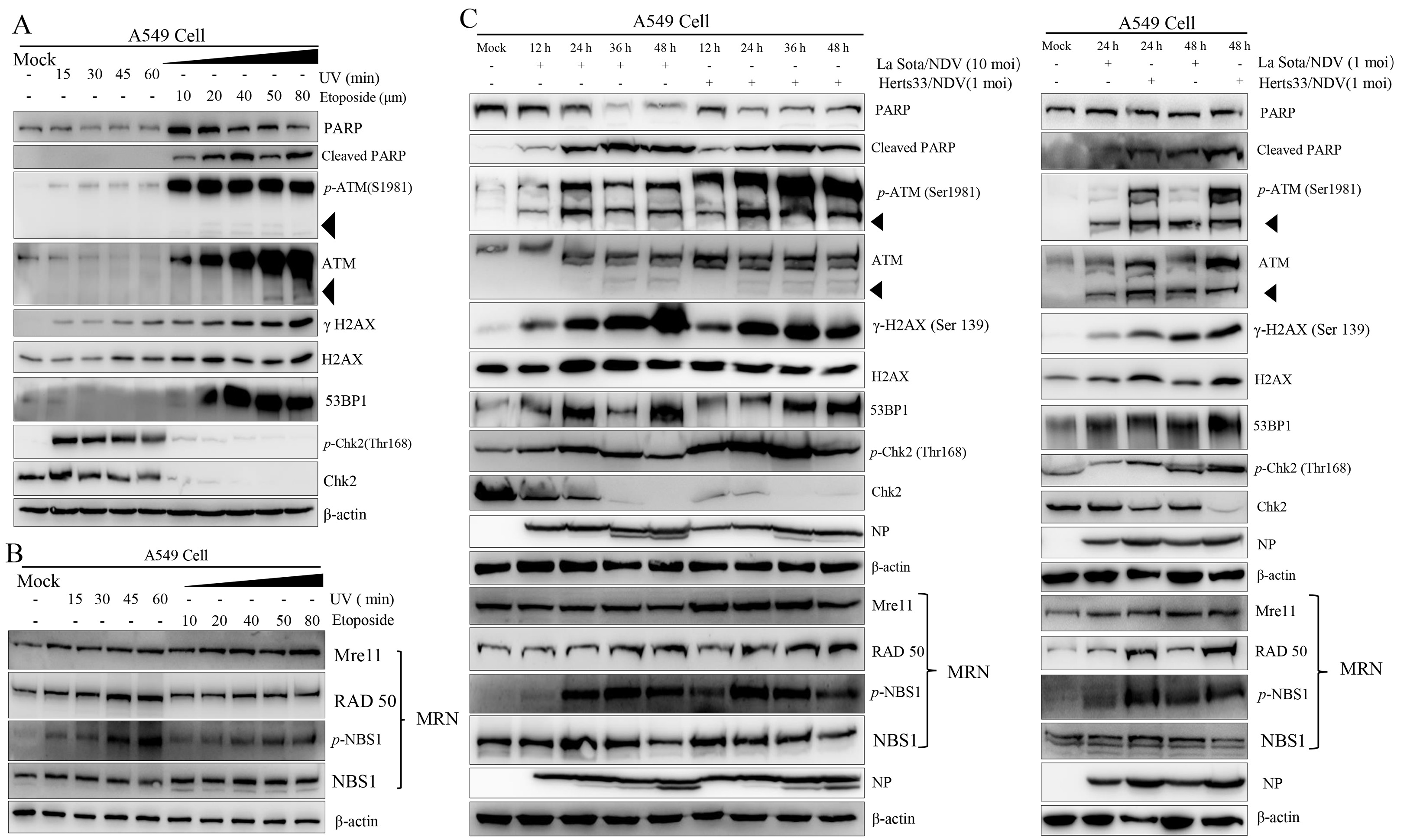

Supplement: S1 Fig — (A) Ultraviolet (UV) exposure and etoposide treatment activated ATM-mediated DSB signaling in A549 cells. Cells treated with UV and etoposide were used as positive controls for inducing DDR. Western blot samples were prepared from A549 cells after UV exposure at 75 mW/cm2 using a low-pressure mercury vapor discharge lamp corresponding to the marked timepoints (15, 30, 45, and 60 min). A549 cells were treated with etoposide at working concentrations of 10, 20, 40, 50, and 80 μm for 24 h. After UV exposure and etoposide treatments, we then conducted Western blot analysis in accordance with the procedures in the Materials and Methods section. The monomer ATM was marked with a black triangle. β-actin served as a loading control. (B) UV and etoposide treatments activated the MRN sensor of ATM-mediated DSB signaling in A549 cells. Cells treated with UV and etoposide served as positive controls for DDR induction. (C) Velogenic NDV and lentogenic NDV strains activated the ATM-mediated DSB signaling in A549 cells. Western blot samples were prepared from A549 cells with lentogenic NDV infection (La Sota strain, MOI = 1 or 10) or virulent NDV (Herts/33 strain, MOI = 1) corresponding to the marked timepoints and analyzed in accordance with the procedures in the Materials and Methods section. (TIF) [file ppat.1008514.s001.tif]

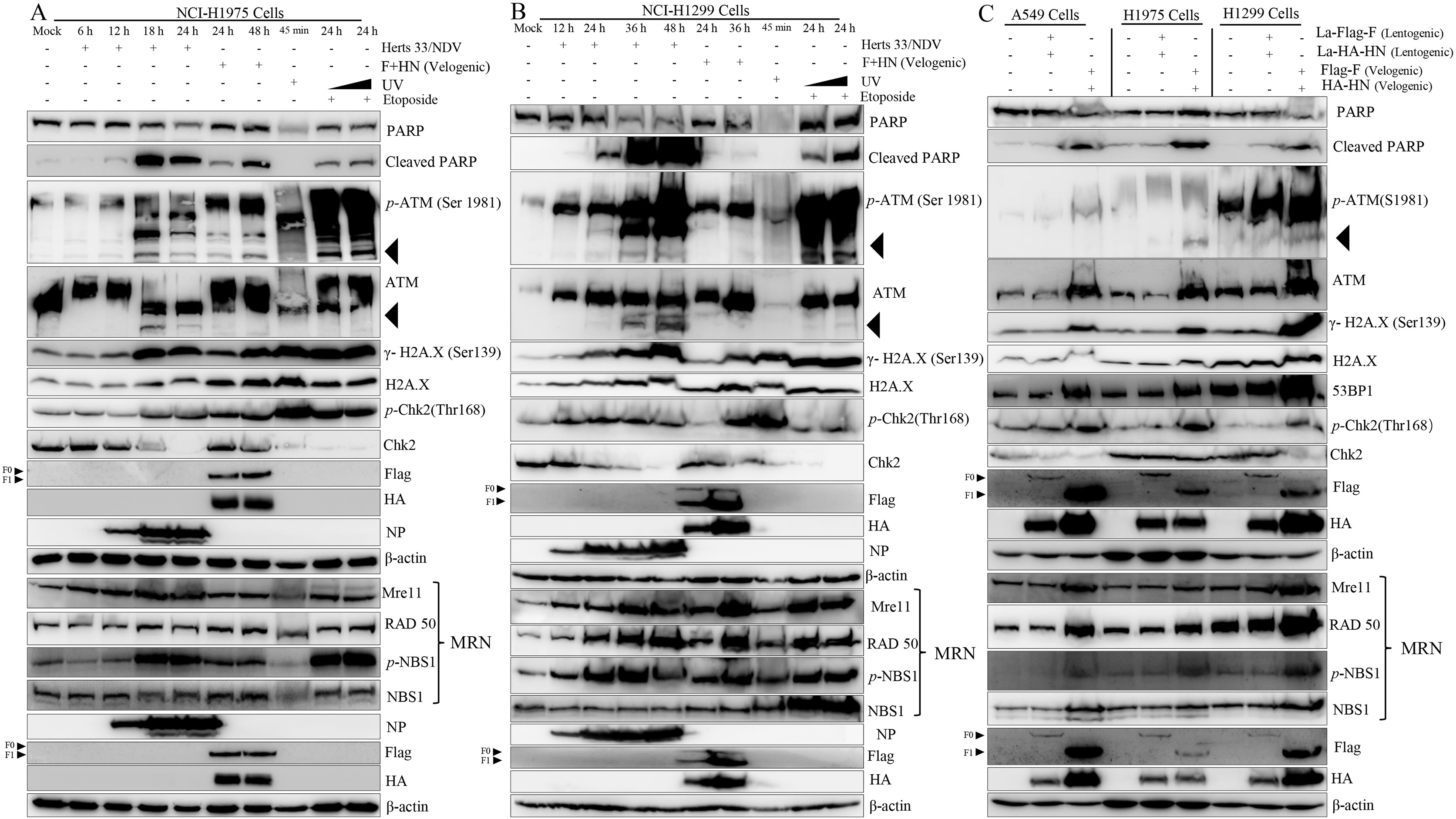

Supplement: S2 Fig — (A) Virulent NDV infection and membrane fusion activated ATM-mediated DSB signals and MRN complex signals in NCI-H1975 cells as discovered by Western blot analysis. Samples were prepared from NCI-H1975 cells after virulent oncolytic NDV infection (Herts/33 strain, MOI = 1) corresponding to the marked timepoints, UV-exposed for 45 min, and treated with etoposide at a final concentration of 80 μm for 24 h, and then co-transfected with both Flag-F and HA-HN plasmids for 24 h and 48 h. Cells treated with UV and etoposide were used as a positive controls for DDR induction. The monomer ATM was marked with a black triangle. (B) Virulent NDV infection and membrane fusion activated ATM-mediated DSB signals and MRN complex signals in NCI-H1299 cells as discovered by Western blot analysis. Samples were prepared from NCI-H1299 cells after virulent oncolytic NDV infection (Herts/33 strain, MOI = 1) corresponding to the marked timepoints, UV-exposed for 45 min, and treated with etoposide at a final concentration of 80 μm for 24 h, and then co-transfected with both Flag-F and HA-HN plasmids for 24 h and 36 h. (C) Membrane fusion triggered by F and HN of velogenic NDV activated ATM-mediated DSBs signal in A549, NCI-H1975, and NCI-H1299 cells as discovered by Western blot analysis. A549, NCI-H1975, and NCI-1299 cells were mock-transfected or co-transfected with both La-Flag-F and La-HA-HN plasmids or both Flag-F and HA-HN plasmids for 36 h. (TIF) [file ppat.1008514.s002.tif]

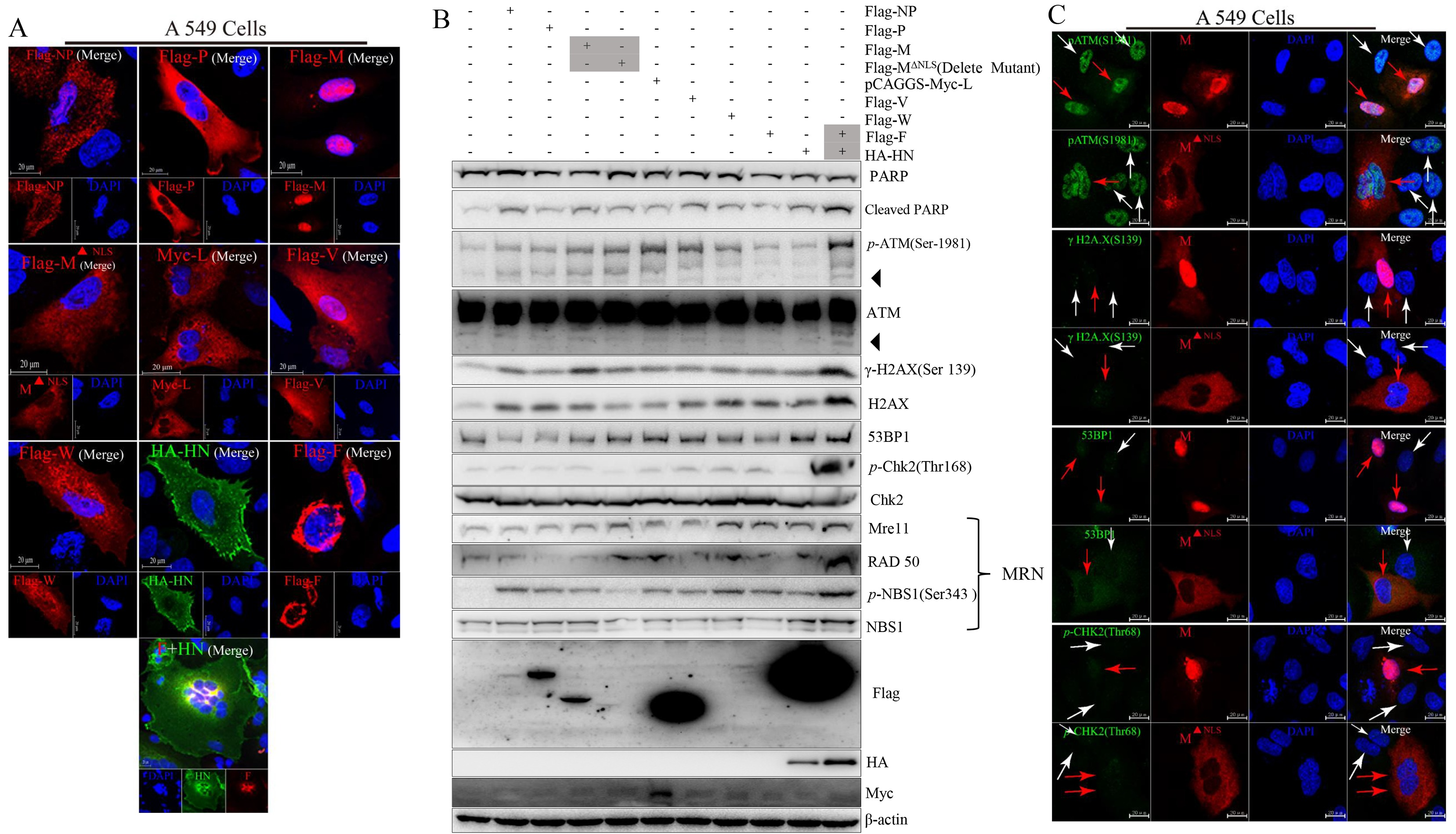

Supplement: S3 Fig — (A) Subcellular localization of structural and non-structural protein of virulent oncolytic NDV in A549 cells. A549 cells were transfected with Flag-NP, Flag-P, Flag-M, Flag-MΔNLS, pCAGGS-Myc-L, Flag-V, Flag-W, HA-HN, Flag-F, and F-HN for 24 h in A549 cells. Flag-Tag (Red); nuclei (blue); HA-Tag (Green). Scale bars = 20 μm. (B) Synergistic cooperation of F and HN activated ATM-dependent DSBs as discovered by Western blot analysis. A549 cells were mock-transfected or transfected with Flag-NP, Flag-P, Flag-M, Flag-MΔNLS, pCAGGS-Myc-L, Flag-V, Flag-W, HA-HN, Flag-F, and F-HN for 36 h. After transfection, we then conducted Western blot analyzed in accordance with the procedures in the Materials and Methods section. The monomer ATM was marked with a black triangle. (C) The structural M protein of NDV did not activate the ATM-mediated DSBs pathway in A549 cells. A549 cells were transfected with Flag-M and Flag-MΔNLS for 36 h. p-ATM (Ser 1981), γ-H2A.X (Ser 139), and p-Chk2 (Thr 48) were stained with green, nuclei with blue, Flag-tag with green. The red arrow indicates the transfected cells and black arrow indicates mock-transfected cells used as a itself control. (TIF) [file ppat.1008514.s003.tif]

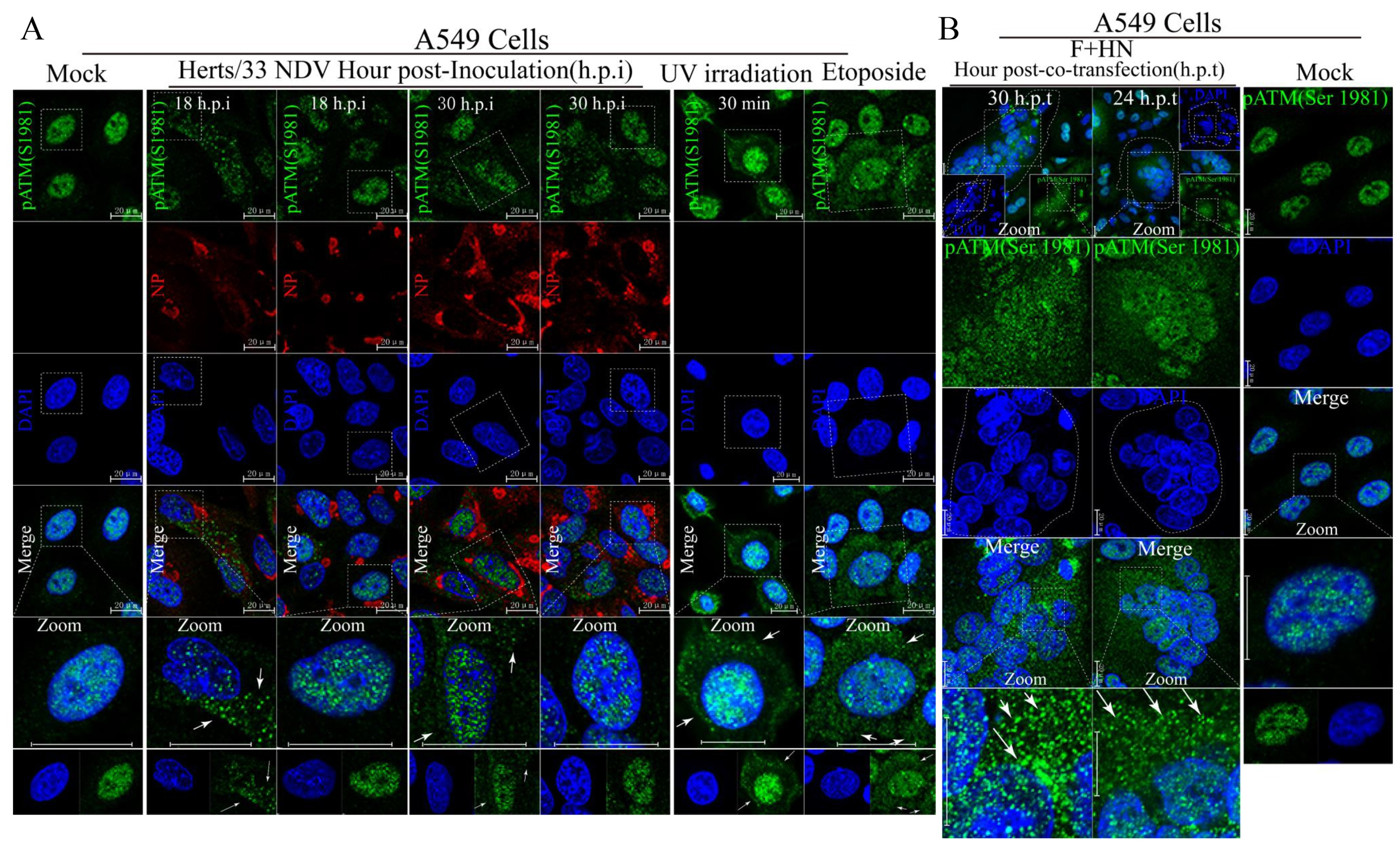

Supplement: S4 Fig — (A) Subcellular localization of ATM phosphorylation on Ser1981 in response to virulent oncolytic NDV infection in A549 cells. A549 cells were mock-infected and NDV-infected (Herts/33, MOI = 1) for 18 h and 30 h, UV exposed for 30 min, and treated with etoposide at a final concentration of 80 μm for 24 h. Phosphorylated ATM (green); nuclei (blue); NDV (red). The arrow indicates localization of phosphorylated ATM in cytoplasm. Scale bars = 20 μm. (B) Subcellular localization of endogenous ATM phosphorylation on Ser1981 in response to F-HN co-expression in A549 cells. A549 cells were mock-transfected or co-transfected with both Flag-F and HA-HN plasmids for 24 h and 30 h. (TIF) [file ppat.1008514.s004.tif]

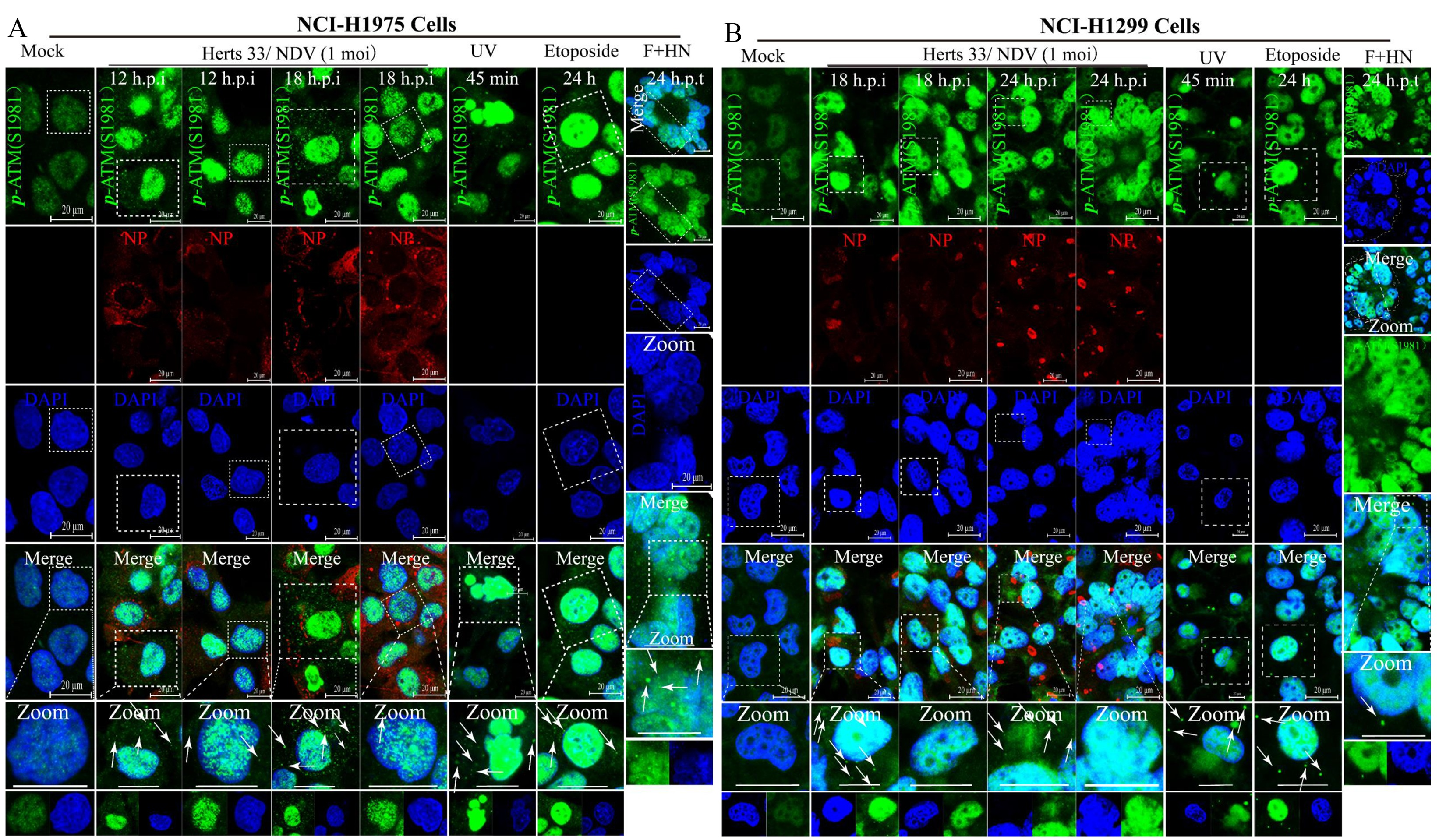

Supplement: S5 Fig — (A) Subcellular localization of ATM phosphorylation on Ser1981 in response to virulent oncolytic NDV infection and F-HN co-expression in NCI-H1975 cells. NCI-H1975 cells were mock-infected and NDV-infected (Herts/33, MOI = 1) for 12 h and 18 h, UV-exposed for 45 min, and treated with etoposide at a final concentration of 80 μm for 24 h. The UV and etoposide treatment groups served as the positive controls. NCI-H1975 cells were co-transfected with both Flag-F and HA-HN plasmids for 24 h. Phosphorylated ATM (green); nuclei (blue); NDV (red). The arrow indicates localization of phosphorylated ATM in cytoplasm. Scale bars = 20 μm. (B) Subcellular localization of ATM phosphorylation on Ser1981 in response to virulent oncolytic NDV infection and F-HN co-expression in NCI-H1299 cells. NCI-H1299 cells were mock-infected and NDV-infected (Herts/33, MOI = 1) for 18 h and 24 h, UV-exposed for 45 min, treated with etoposide at a final concentration of 80 μm for 24 h, and then co-transfected with both Flag-F and HA-HN plasmids for 24 h. (TIF) [file ppat.1008514.s005.tif]

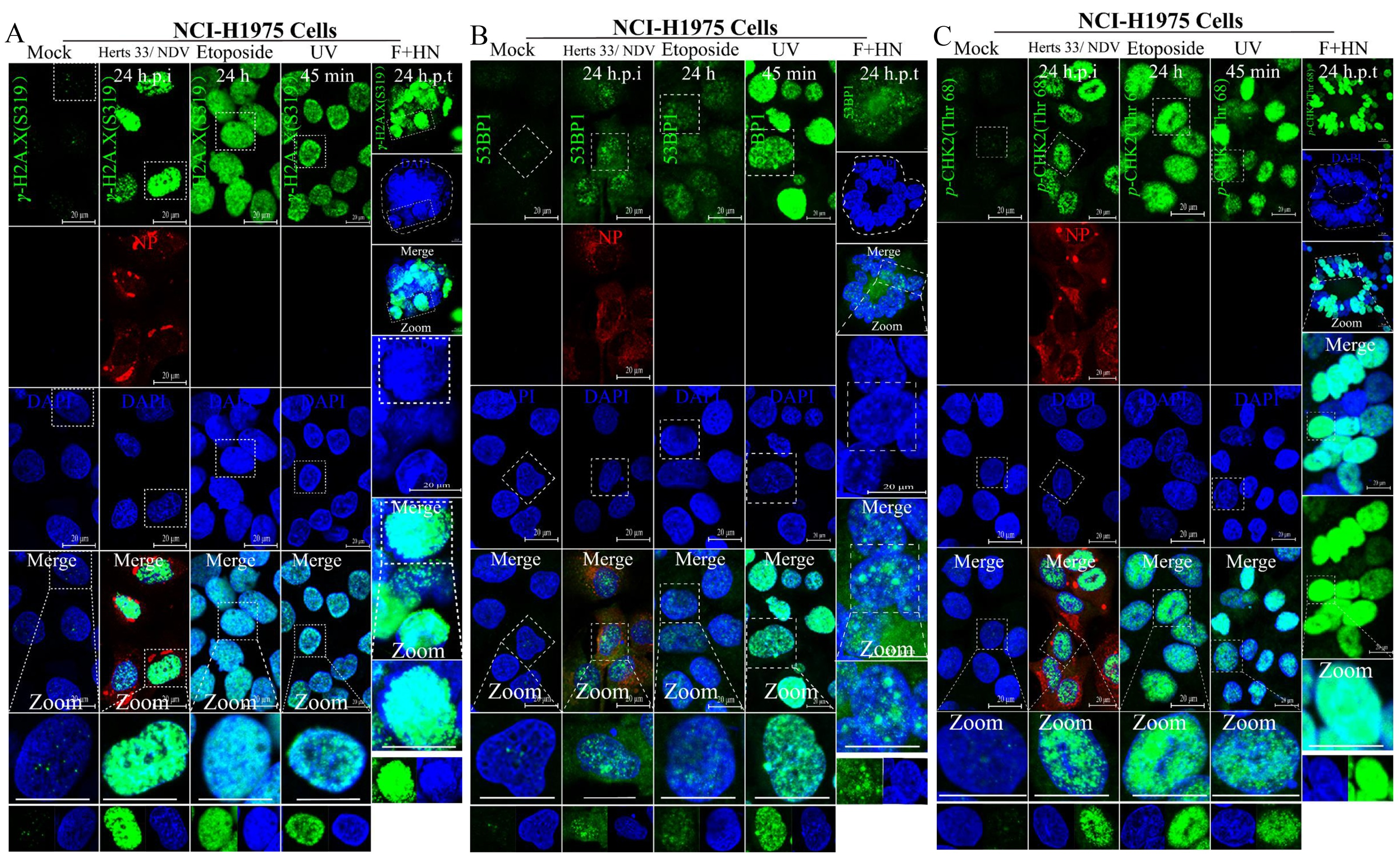

Supplement: S6 Fig — (A) Representative images showing that virulent NDV infection and F-HN co-expression triggered the nuclear aggregation of γ-H2A.X in NCI-H1975 cells. NCI-H1975 cells were mock-infected, NDV-infected (MOI = 1), inoculated for 24 h, UV-exposed for 45 min, treated with etoposide at a final concentration of 80 μm for 24 h, and then mock-transfected or co-transfected with both Flag-F and HA-HN plasmids for 24 h. The UV and etoposide treatment groups served as the positive controls. γ-H2AX (green); nuclei (blue); NDV (red). Scale bars = 20 μm. (B) Representative images showing that virulent NDV infection and F-HN co-expression triggered nuclear aggregation of 53BP-1 in NCI-H1975 cells. NCI-H1975 cells were mock-infected, NDV-infected (MOI = 1), inoculated for 24 h, UV-exposed for 45 min, treated with etoposide at a final concentration of 80 μm for 24 h, and then mock-transfected or co-transfected with both Flag-F and HA-HN plasmids for 24 h. 53BP1 (green); nuclei (blue); NDV (red). (C) Representative images showing that virulent NDV infection and F-HN co-expression triggered nuclear aggregation of p-Chk2 in NCI-H1975 cells. NCI-H1975 cells were mock-infected, NDV-infected (MOI = 1), inoculated for 24 h, UV-exposed for 45 min, and treated with etoposide at a final concentration of 80 μm for 24 h, and then mock-transfected or co-transfected with both Flag-F and HA-HN plasmids for 24 h. Phosphorylated Chk2 (green); nuclei (blue); NDV (red). (TIF) [file ppat.1008514.s006.tif]

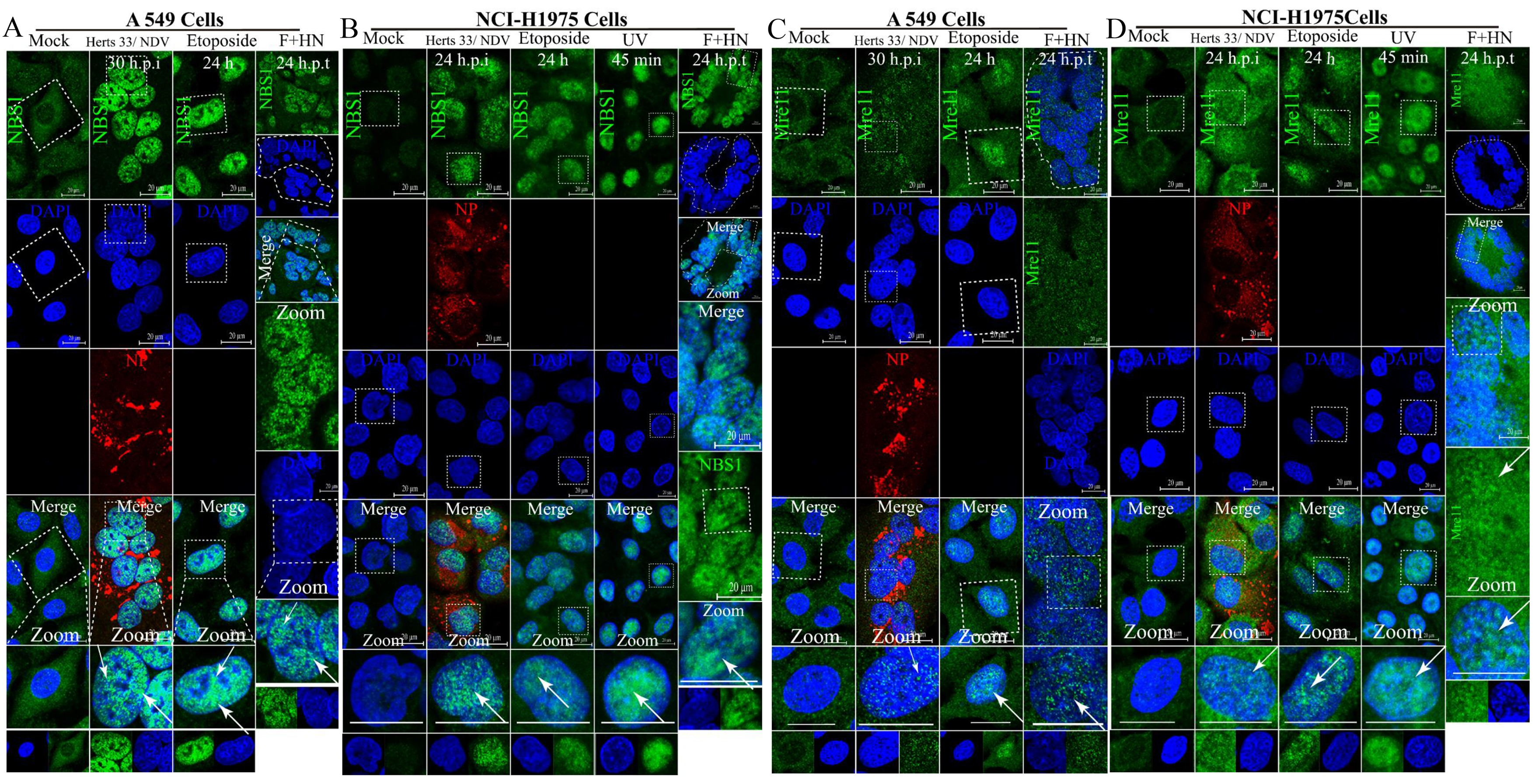

Supplement: S7 Fig — (A) Representative images showing the spatial redistribution of NBS1 in response to virulent oncolytic NDV infection and F and HN co-expression in A549 cells. A549 cells were mock-infected or NDV-infected (MOI = 1) for 30 h, treated with etoposide at a final concentration of 80 μm for 24 h, and then mock-transfected or co-transfected with both Flag-F and HA-HN plasmids for 24 h. NBS1 (green); nuclei (blue); NDV (red). The etoposide treatment group served as the positive control. The arrow indicates the localization of NBS1 in the nucleus. Scale bars = 20 μm. (B) Representative images showing the spatial redistribution of NBS1 in response to virulent oncolytic NDV infection and F and HN co-expression in NCI-H1975 cells. NCI-H1975 cells were mock-infected and NDV-infected (MOI = 1) for 24 h, UV-exposed for 45 min, treated with etoposide at a final concentration of 80 μm for 24 h, and then mock-transfected or co-transfected with both Flag-F and HA-HN plasmids for 24 h. NBS1 (green); nuclei (blue); NDV (red). (C) Representative images showing the spatial redistribution of Mre11 in response to virulent oncolytic NDV infection and F and HN co-expression in A549 cells. A549 cells were mock-infected and NDV-infected (MOI = 1) for 30 h, treated with etoposide at a final concentration of 80 μm for 24 h, and then mock-transfected or co-transfected with both Flag-F and HA-HN plasmids for 24 h. Mre11 (green); nuclei (blue); NDV (red). (D) Representative images showing the spatial redistribution of Mre11 in response to virulent oncolytic NDV infection and F and HN co-expression in NCI-H1975 cells. NCI-H1975 cells were mock-infected and NDV-infected (MOI = 1) for 24 h, UV exposed for 45 min, treated with etoposide at a final concentration of 80 μm for 24 h, and then mock-transfected or co-transfected with both Flag-F and HA-HN plasmids for 24 h. Mre11 (green); nuclei (blue); NDV (red). (TIF) [file ppat.1008514.s007.tif]

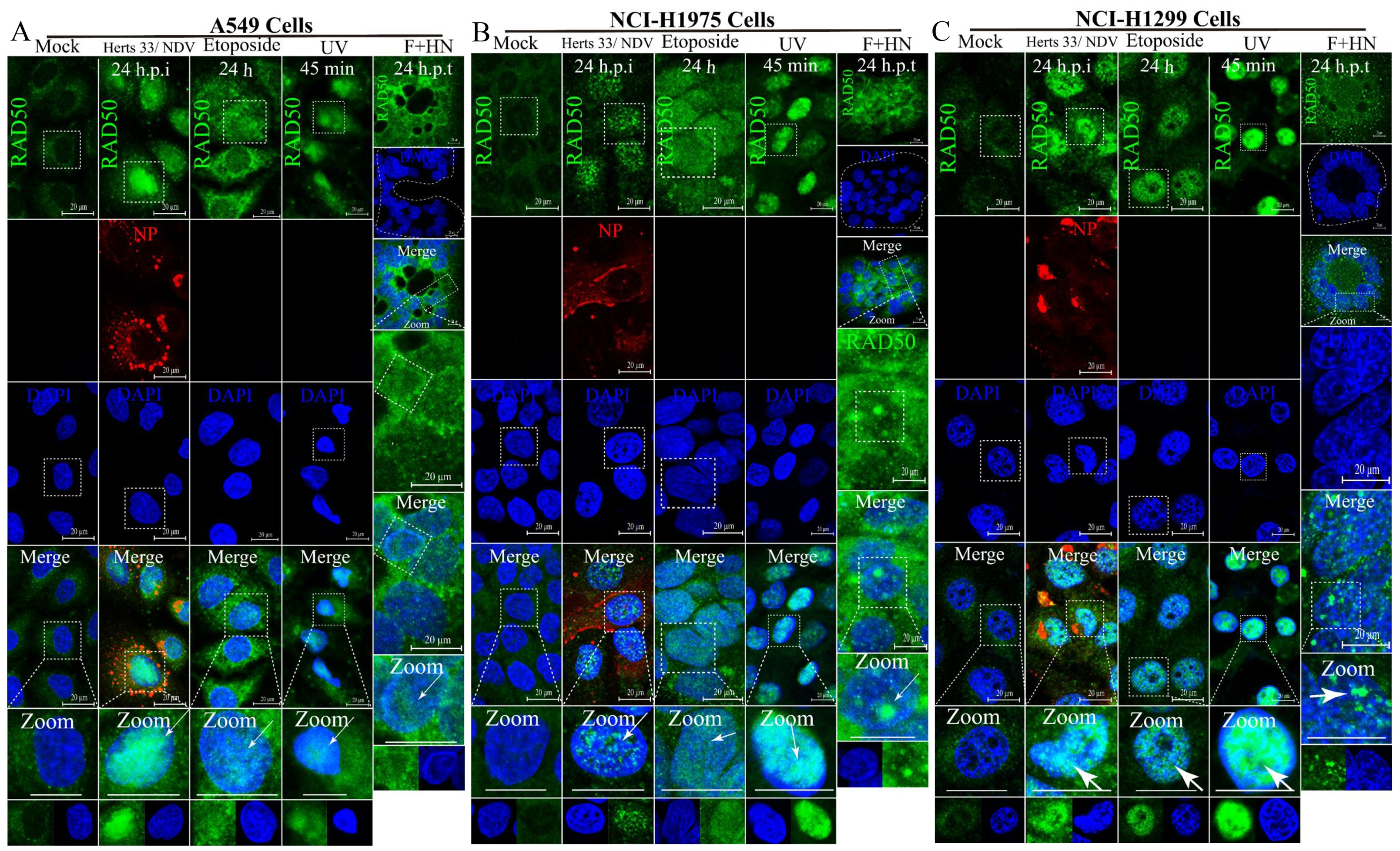

Supplement: S8 Fig — (A) Representative images showing the spatial redistribution of Rad50 in response to virulent oncolytic NDV infection and F and HN co-expression in A549 cells. A549 cells were mock-infected and NDV-infected (MOI = 1) for 24 h, and treated with etoposide at a final concentration of 80 μm for 24 h, and then mock-transfected or co-transfected with both Flag-F and HA-HN plasmids for 24 h. Rad50 (green); nuclei (blue); NDV (red). The etoposide treatment and UV-groups served as the positive controls. The arrow indicates localization of NBS1in the nucleus. Scale bars = 20 μm. (B) Representative images showing the spatial redistribution of Rad50 in response to virulent oncolytic NDV infection and F and HN co-expression in NCI-H1975 cells. NCI-H1975 cells were mock-infected and NDV-infected (MOI = 1) for 24 h, treated with etoposide at a final concentration of 80 μm for 24 h, and then mock-transfected or co-transfected with both Flag-F and HA-HN plasmids for 24 h. Rad50 (green); nuclei (blue); NDV (red). (C) Representative images showing the spatial redistribution of Rad50 in response to virulent oncolytic NDV infection and F and HN co-expression in NCI-H1299 cells. NCI-H1299 cells were mock-infected and NDV-infected (MOI = 1) for 24 h, and treated with etoposide at a final concentration of 80 μm for 24 h, and then mock-transfected or co-transfected with both Flag-F and HA-HN plasmids for 24 h. Rad50 (green); nuclei (blue); NDV (red). (TIF) [file ppat.1008514.s008.tif]

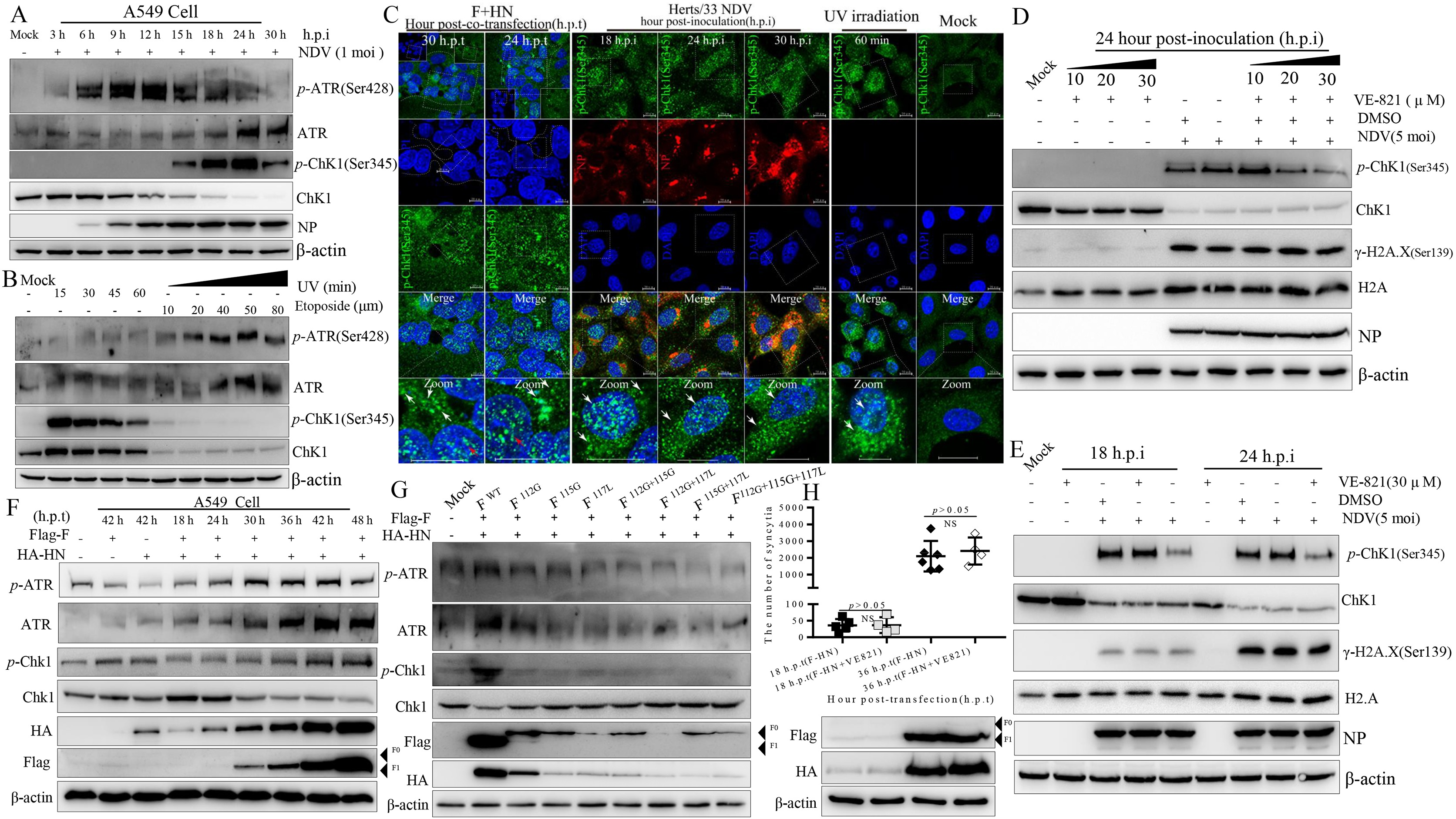

Supplement: S9 Fig — (A) Virulent NDV infection activated ATR kinase as discovered by Western blot analysis. Samples were prepared from A549 cells after virulent oncolytic NDV infection (Herts/33 strain, MOI = 1) corresponding to the marked timepoints and analyzed in accordance with the procedures in the Materials and Methods section. β-actin served as a loading control. (B) UV exposure and etoposide treatment activated ATR kinase in A549 cells. Cells treated with UV and etoposide served as positive controls. Western blot samples were prepared from A549 cells after UV-exposure corresponding to the marked timepoints (15, 30, 45, and 60 min). A549 cells were treated with etoposide at working concentrations of 10, 20, 40, 50, and 80 μm for 24 h. (C) Representative images showing that virulent oncolytic NDV infection and F-HN co-expression triggered the nuclear aggregation of phosphorylated Chk1 on Ser345 in A549 cells. A549 cells were mock-infected or NDV-infected (MOI = 1) for 18, 24, and 30 h, UV exposed for 60 min, treated with etoposide at a final concentration of 80 μm for 24 h, and then mock-transfected or co-transfected with both Flag-F and HA-HN plasmids at the indicated timepoint. At 24 and 30 h.p.t., coverslips were examined by IFA. The UV and etoposide treatment groups served as the positive controls. p-Chk1 (green); nuclei (blue); NDV (red). Scale bars = 20 μm. (D) ATR kinase was not required for NDV intracellular replication in a dose-dependent manner. A549 cells were pretreated at a working concentration of VE-821 at 10, 20, and 30 μm for 1 h prior to NDV infection, and mock-infected, NDV-infected (MOI = 5) for 24 h. (E) ATR kinase was not required for NDV intracellular replication in a time-dependent manner. A549 cells were pretreated at a working concentration of VE-821 at 30 μm for 1 h prior to NDV infection, and mock-infected and NDV-infected (MOI = 5) for 18 and 24 h. (F) F and HN cooperated synergistically to activate ATR kinase in A549 cells. A549 cells were co-transfe [file ppat.1008514.s009.tif]
